# Physiological and transcriptomic analysis uncovers salinity stress mechanisms in a facultative crassulacean acid metabolism plant *Dendrobium officinale*

**DOI:** 10.3389/fpls.2022.1028245

**Published:** 2022-10-06

**Authors:** Mingze Zhang, Nan Liu, Jaime A. Teixeira da Silva, Xuncheng Liu, Rufang Deng, Yuxian Yao, Jun Duan, Chunmei He

**Affiliations:** ^1^ The Department of Life Science and Agriculture, Qiannan Normal University for Nationalities, Duyun, China; ^2^ Key Laboratory of South China Agricultural Plant Molecular Analysis and Genetic Improvement, Provincial Key Laboratory of Applied Botany, South China Botanical Garden, Chinese Academy of Sciences, Guangzhou, China; ^3^ Key Laboratory of Vegetation Restoration and Management of Degraded Ecosystems, South China Botanical Garden, Chinese Academy of Sciences, Guangzhou, China; ^4^ Independent Researcher, Kagawa-ken, Japan; ^5^ Opening Public Laboratory, Chinese Academy of Sciences, Guangzhou, China; ^6^ Center of Economic Botany, Core Botanical Gardens, Chinese Academy of Sciences, Guangzhou, China

**Keywords:** *Dendrobium officinale*, differentially expressed genes, metabolic adjustment, pigment biosynthesis, plant hormones signaling

## Abstract

*Dendrobium officinale* is a precious medicinal Chinese herb that employs facultative crassulacean acid metabolism (CAM) and has a high degree of abiotic stress tolerance, but the molecular mechanism underlying the response of this orchid to abiotic stresses is poorly understood. In this study, we analyzed the root microstructure of *D*. *officinale* plantlets and verified the presence of chloroplasts by transmission electron microscopy. To obtain a more comprehensive overview of the molecular mechanism underlying their tolerance to abiotic stress, we performed whole‐transcriptome sequencing of the roots of 10-month-old plantlets exposed to salt (NaCl) treatment in a time‐course experiment (0, 4 and 12 h). The total of 7376 differentially expressed genes that were identified were grouped into three clusters (*P* < 0.05). Metabolic pathway analysis revealed that the expression of genes related to hormone (such as auxins, cytokinins, abscisic acid, ethylene and jasmonic acid) biosynthesis and response, as well as the expression of genes related to photosynthesis, amino acid and flavonoid metabolism, and the SOS pathway, were either up- or down-regulated after salt treatment. Additionally, we identified an up-regulated WRKY transcription factor, *DoWRKY69*, whose ectopic expression in Arabidopsis promoted seed germination under salt tress. Collectively, our findings provide a greater understanding of the salt stress response mechanisms in the roots of a facultative CAM plant. A number of candidate genes that were discovered may help plants to cope with salt stress when introduced *via* genetic engineering.

## Introduction

Plants may face adverse environmental conditions, such as extreme temperatures, drought, and salinity are major abiotic stressors, that hamper their metabolism, delay growth and development, reduce productivity, or even cause plant death (Krasensky and Jonak, 2012; Raza et al., 2022a). High salinity strongly and negatively influences the productivity of agricultural crops by impacting seed germination and plant vegetative growth, and stimulates osmotic and oxidative stress, as well as ion toxicity (Deinlein et al., 2014).

However, under salt stress, plants can decrease ion toxicity and scavenge reactive oxygen species (ROS) by regulating ion homeostasis, induce antioxidant defense systems, and synthesize a variety of plant hormones and osmoprotectants (Raza et al., 2022a). Salinity can impact plants rapidly by inducing osmotic stress, or slowly by inducing ionic stress (Munns and Tester, 2008). In addition, salinity can disrupt the integrity of the chloroplast envelope, leading to the disorganization of grana, resulting in a reduced photosynthetic rate that can be partly attributed to the reduction of photosynthetic pigments (Kwon et al., 2019). To allow plants to cope with salt stress, signal transduction pathways such as phytohormone-mediated and Ca^2+^ signaling pathways are activated (Knight et al., 1997; Zhu, 2002; Kaleem et al., 2018). Under high salinity stress, plants modify their levels of endogenous hormones such as gibberellins (GAs), abscisic acid (ABA) and jasmonic acid (JA), thereby activating the genes involved in the metabolism of those hormones to cope with this stress (Kaleem et al., 2018). GA is involved in salt stress response pathways by negatively regulating a class of DELLA proteins (Verma et al., 2016). ABA promotes stomatal closure to reduce water loss by rapidly altering ion fluxes in guard cells under osmotic stress (Munemasa et al., 2015). Most studies have illustrated the role of JA in response to biotic stresses, while evidence of the involvement of JA in salinity stress has only emerged over the last decade (Kazan, 2015; Wasternack, 2015; Abouelsaad and Renault, 2018). In addition, auxins and cytokinins mediate stress-adaptation responses in plants (Bielach et al., 2017).

Some studies have demonstrated that hormone-mediated signal transduction pathways trigger transcription factors (TFs) such as members of the bZIP, NAC, ERF, WRKY, MYB, ZF-HD, and bHLH families to amplify signals by inducing or repressing functional genes, thereby initiating protective mechanisms that allow plants to cope with salinity stress (Deinlein et al., 2014; Kaleem et al., 2018; Shalmani et al., 2019; Mansour and Hassan, 2022). For example, the dehydration-responsive element binding/C-repeat-binding factor (DREB/CBF), which is a member of the AP2/ERF TF subfamily, can modulate drought-, high temperature-, salt-, and cold-responsive gene expression (Dubouzet et al., 2003). WRKY TFs are involved in the response of plants to salinity stress. In *Arabidopsis thaliana*, *AtWRKY46* plays a role in regulating stomatal opening, with mutant *wrky46* plants showing sensitivity to drought and salt stress (Ding et al., 2015). The interaction between GmWRKY27 and GmMYB174 proteins from *Glycine max* regulates *GmNAC29* expression, improving salt and drought tolerance (Wang et al., 2015b). *CgWRKY57*, which was identified in an orchid (*Cymbidium goeringii*), sensed ABA signals and might play a role in stress response (Liu et al., 2021). Overexpression of *WRKY* genes from *Dendrobium nobile* enhanced salt and stress tolerance in transgenic tobacco (Xu et al., 2014). Compatible solutes, such as proline, glycine betaine, sugars, mannitol and polyols, may accumulate in the cytoplasm, allowing water potential to be adjusted and cell turgor to be maintained under salinity stress (Aziz and Larher, 1995; Krasensky and Jonak, 2012; Moharramnejad et al., 2015). In *Medicago sativa*, most *MsZF-HDs* responded to salt stress (He et al., 2022). In *A. thaliana*, MYC2, which is a bHLH TF, negatively regulated proline biosynthesis by repressing *P5CS1* expression in response to salt stress (Verma et al., 2020). Moreover, an R2R3-MYB TF-encoding gene *IbMYB308* from sweet potato (*Ipomoea batatas*) improve the salt tolerance of transgenic tobacco (Wang et al., 2022).

The Orchidaceae is the most diverse flowering plant family and includes approximately 880 genera and 28,000 species around the world (Givnish et al., 2015). *Dendrobium*, which is the second largest genus in the Orchidaceae, has adapted to diverse natural habitats, ranging from high altitudes to lowland tropical forests, and even to the dry climate of the Australian desert, implying its strong adaptability to adverse environmental conditions. *Dendrobium* plants are known to survive under high salinity (15 dS m^−1^) (Abdullakasim et al., 2018), indicating that they display high endurance to salinity stress. *Dendrobium officinale* is a precious Chinese medicinal herbal orchid with various bioactivities (Teixeira da Silva and Ng, 2017). A previous study by our group showed that *D*. *officinale* could adapt to high concentrations of salt stress, and in doing so, accumulated bioactive metabolites (Zhang et al., 2021).

Although a tremendous amount of research has been conducted on salt stress in model plants and crops, known strategies for coping with salt stress in orchids is not commensurate with their recalcitrant nature. Understanding the mechanisms by which orchid plants cope with the challenge of salt stress will pave a way for planning future initiatives for abiotic (salt) stress engineering in orchids to protect orchid plants in the wild. In this study, we investigated changes to the transcriptome under short-term (within 12 h) salinity stress in the roots of *D*. *officinale* plantlets. We identified differentially expressed genes (DEGs) and focused on phytohormone signaling, the SOS pathway, photosynthesis and metabolic adjustments such as changes in amino acid content and flavonoid metabolism. We also identified differentially expressed TFs and characterized the role of a single *WRKY* gene in seed germination under salinity stress. Our results provide new insight into the biochemical and molecular regulation of salt stress tolerance mechanisms of orchid plants that enhance their stress tolerance.

## Materials and methods

### Plant growth conditions and treatments

*D*. *officinale* plantlets that were geminated from seeds within the same capsule, were grown *in vitro* on half-strength Murashige and Skoog (MS) (Murashige and Skoog, 1962) medium containing 2% sucrose and 0.6% agar (pH 5.4) in a growth chamber (25 ± 1°C, 40 µmol m^-2^ s^-1^, 12-h photoperiod). Uniform plantlets 8-9 cm in height were transferred to half-strength MS containing 2% sucrose and 0.6% agar, and supplemented with 250 mM NaCl (pH 5.4) and kept at 25 ± 1°C, under 40 µmol m^-2^ s^-1^, in a 12-h photoperiod. Root samples were collected at three time points [0 (control), 4 and 12 h], frozen in liquid nitrogen and used to isolate RNA. Three independent biological replicates were used for each time point and six plantlets were used for each replicate. To analyze chlorophyll (Chl) and carotenoids, plantlets were subjected to salinity treatment or not (the control) for two weeks, and roots were harvested and used to detect these pigments. To analyze total flavonoids and free amino acids, roots were collected after two weeks from control and salt-treated plants and freeze dried. Root samples were ground to a fine powder by a RETSCH MM400 Mixer Mill (Retsch Technology, Haan, Germany). Three independent biological replicates were utilized for each sample.

### Histological analysis of *D. officinale* plantlet roots

The roots of plantlets 8-9 cm in height were collected and fixed in fixation buffer [2.5% glutaraldehyde and 2% paraformaldehyde in 0.1 M sodium phosphate buffer (pH 7.2)]. To facilitate penetration of the fixative, samples were vacuum infiltrated for at least 30 min. Root samples were rinsed in wash buffer (1% sodium phosphate) six times (30 min each time) after fixation, then fixed in a 0.1 M sodium cacodylate buffer (pH 7.2) containing 1% osmium tetroxide (OsO_4_) for 4 h. An ethanol concentration gradient (30, 50, 75, 85, 95, 100%, v/v) was used to dehydrate samples. After dehydration, samples were treated in different ratios of acetone and Epon812 (3:1, 1:1 and 1:3, v/v) for 30 min. Finally, samples were embedded in absolute Epon812 overnight and placed at 60°C for two days. Transverse sections of root samples were cut into slices of 1 μm thickness with an LKB-11800 ultramicrotome and stained by periodic acid Schiff (PAS) (Tütüncü Konyar et al., 2013). Root samples were cut into 50-70 nm thick slices with a LeicaUC6 ultramicrotome for observations using a JEOL JEM 1010 (JEOL Ltd., Tokyo, Japan) transmission electron microscope.

### Transcriptomic analysis

Total RNA from root samples at 0 (control), 4 and 12 h after salinity stress was isolated using Column Plant RNAout2.0 (Tiandz Inc., Beijing, China). Biological triplicates for each time point were used for RNA sequence (RNA-seq) analysis in this study. The mRNA of each sample was purified from total RNA using oligo d(T)_25_ magnetic beads (New England BioLabs Inc., Ipswich, MA, USA). A library of the isolated mRNA was prepared with the NEBNext^®^ Ultra™ RNA Library Prep Kit (New England Biolabs Inc.) then subjected to paired-end sequencing with the Illumina Novaseq 6000 Sequencing System at Biomarker Technologies Inc. (Beijing, China). The raw reads produced from sequencing were processed through in-house perl scripts (Biomarker Technologies Inc.) to remove reads containing an adapter, ploy-N or poor-quality reads (the quality Q ≤ 30 accounted for more than 50% of all reads). The remaining reads were clean reads, which were mapped with the *D*. *officinale* version 2 genome generated by Zhang et al. (2017) using TopHat version 2.0.8 (Kim et al., 2013a). About 90% of clean reads were mapped to the *D*. *officinale* genome. We performed differential expression analysis of RNA-seq data by comparing the expression of genes in treatments (4 h and 12 h) and the control (0 h) by using DESeq2 (Love et al., 2014). Genes with a fold change (treatment/control) ≥ 2.0 and a false discovery rate (FDR) ≤ 0.01 were regarded as up-regulated genes while genes with a fold change (treatment/control) ≤ 0.5 and an FDR ≤ 0.01 were regarded as down-regulated genes. All the up- and down-regulated genes were defined as DEGs. The clean reads generated in this study were submitted to the Sequence Read Archive (SRA) database of the National Center for Biotechnology (NCBI) under the following accession numbers: SRS8480001, SRS8480000 and SRS8480010 for 0 h; SRS8480011, SRS8480012 and SRS8480013 for 4 h; SRS8480016, SRS8480004 and SRS8480002 for 12 h.

### Functional annotation of genes

Seven public gene functional annotation databases, NCBI non-redundant protein sequences database (Nr, http://www.ncbi.nlm.nih.gov), Protein family (Pfam, http://pfam.xfam.org/) (Finn et al., 2013), Clusters of Orthologous Groups of proteins (COG, http://www.ncbi.nlm.nih.gov/COG) (Galperin et al., 2021), a manually annotated and reviewed protein sequence database (Swiss-Prot, https://www.expasy.org/) (The UniProt Consortium, 2017), Gene Ontology (GO, http://geneontology.org/) (Ashburner et al., 2000), Kyoto Encyclopedia of Genes and Genomes (KEGG, https://www.kegg.jp/) (Kanehisa et al., 2004) and evolutionary genealogy of genes: Non-supervised Orthologous Groups (eggNOG, http://eggnog.embl.de/) (Huerta-Cepas et al., 2019), were used to annotate gene functions. We identified genes based on functional gene annotation. Information about the metabolic pathway of genes was obtained from KEGG annotation. Based on GO annotation, the number of DEGs assigned to each GO term was calculated and visualized in a diagram. To cluster the DEGs, the Short Time-series Expression Miner (STEM) method in STEM software was used (Ernst and Bar-Joseph, 2006). The maximum number of model profiles was 20, and the maximum unit change in model profiles between time points was 1. KOBAS software (Xie et al., 2011) was used to test the statistical enrichment of DEGs in pathways.

### Reverse transcription PCR and quantitative real-time RT-PCR

Eight-day-old seedlings of wild-type Arabidopsis (Col-0) and transgenic lines were grown in half-strength MS medium supplemented with 1.5% sucrose, 0.8% agar (pH 5.7) and placed under a 16-h photoperiod (100 µmol m^-2^ s^-1^) at 22°C. Total RNA was isolated using Column Plant RNAout2.0 (Tiandz Inc.). RNA samples were used for first-strand cDNA synthesis with the GoScript Reverse Transcription System (Promega, Madison, WI, USA). The cDNA of each sample (400 ng μL^-1^) was used as a template for PCR amplification. The genes were amplified by 30 cycles of 98°C for 10 sec, 55°C for 30 sec and 72°C for 30 sec. PCR products (5 mL) of each sample were surveyed by 1% agarose gel electrophoresis and photographed with a gel imaging system (GenoSens1880, Shanghai Qinxiang Scientific Instrument Co. Ltd., Shanghai, China). Quantitative real-time PCR (qRT-PCR) was performed with the Unique Aptamer™ qPCR SYBR^®^ Green Master Mix (Beijing Novogene Technology Co. Ltd.) in a LightCycler 480 system (Roche, Basel, Switzerland). Primer sets are listed in **Supplementary Table 1**.

### Determination of chlorophyll and total carotenoids in roots

Fresh root samples were homogenized in a mortar with a pestle using silica sand and 80% acetone as the extracting solvent. The homogenized mixture was placed at 4°C for 1 h in the dark before centrifuging at 10,000 rpm for 15 min at 4°C. The supernatant was collected and used immediately to detect absorption at 663.2, 646.8 and 470 nm with a UV-6000 spectrophotometer (Shanghai Metash, Shanghai, China). Chl *a* was calculated as 12.25A_663.2_ – 2.79A_646.8_, Chl *b* was calculated as 21.5A_646.8_ – 5.1A_663.2_, and total carotenoids was calculated as (1000 A_470_ – 1.82 Chl *a* – 85.02 Chl *b*)/198 (Sumanta et al., 2014).

### Amino acid quantification

Root powder (100 mg) was solubilized in 5 mL of 0.01 M HCl. After incubating for 30 min at room temperature, samples were centrifuged at 13,000 rpm for 10 min. The supernatant was mixed with absolute ethyl alcohol (20:80, v/v). The mixture was incubated for 15 min at room temperature, then centrifuged at 13,000 g for 10 min. The extract was dried by evaporation under vacuum by a rotary evaporator (Eyela N-1300V-W, Tokyo Rikakikai Co. Ltd., Tokyo, Japan). HCl (0.01 M) was added to dissolve the isolated free amino acids. After filtering through a 0.22 μm filter membrane (Corning Inc., Corning, NY, USA), amino acid profiles were quantified using an Automatic Amino Acid Analyzer (S 433-D, Sykam GmbH, Eresing, Germany). Ninhydrin-derivatized amino acids were measured at 570 nm and at 440 nm. Amino acid concentrations were reported as mg of amino acid per 100 mg of dry weight. A standard stock solution type pH (for physiological amino acid analysis) (Sykam catalog No. S000031, Sykam GmbH) was used as the standard solution.

### Determination of total flavonoids

A colorimetric method described by Ren et al. (2020) was used to analyze total flavonoid content with rutin solutions (4, 8, 12, 16 and 20 μg/mL) serving as standards. Briefly, powdered samples were extracted with 50% (v/v) methanol in an ultrasonication bath (VCX600, Sonics and Materials Inc., Newtown, CT, USA) for 90 min at room temperature, then centrifuged at 12,000 rpm for 20 min. The supernatant was collected and used to measure absorbance at 360 nm with a UV-6000 spectrophotometer. The calibration standard was 50% (v/v) methanol.

### Generation of transgenic plants and germination assay

The *DoWRKY69* gene was isolated and inserted into the *Nco*I site of the pCAMBIA1302 vector. The validated recombinant vector was transformed into *Agrobacterium tumefaciens* EHA105 (Shanghai Weidi Biotechnology Co. Ltd, Shanghai, China) by the freeze-thaw method (Weigel and Glazebrook, 2006) and then transformed into *A. thaliana* by the floral dip method (Clough and Bent, 1998). Seeds of both wild type (WT) and transgenic lines were surface-disinfected then seeded on half-strength MS medium containing 1.5% sucrose, 0.8% agar (pH 5.7), and different concentrations of NaCl (150, 200, and 250 mM). Medium without NaCl served as the control. Seeds were kept at 4°C in the dark for 2 d, then transferred to a 16-h photoperiod (100 µmol m^-2^ s^-1^) at 22°C. When the radicle emerged from the testa, a seed was considered to have germinated. About 60 seeds of each genotype were used and the entire experiment was repeated in triplicate.

### Statistical analyses

Statistical analyses were performed with SigmaPlot12.5 software (Systat Software Inc., San Jose, CA, USA). The Dunnett test (*P* < 0.05) was used to indicate statistically significant differences.

## Results

### Anatomical traits of *D*. *officinale* roots

**Figure 1:**
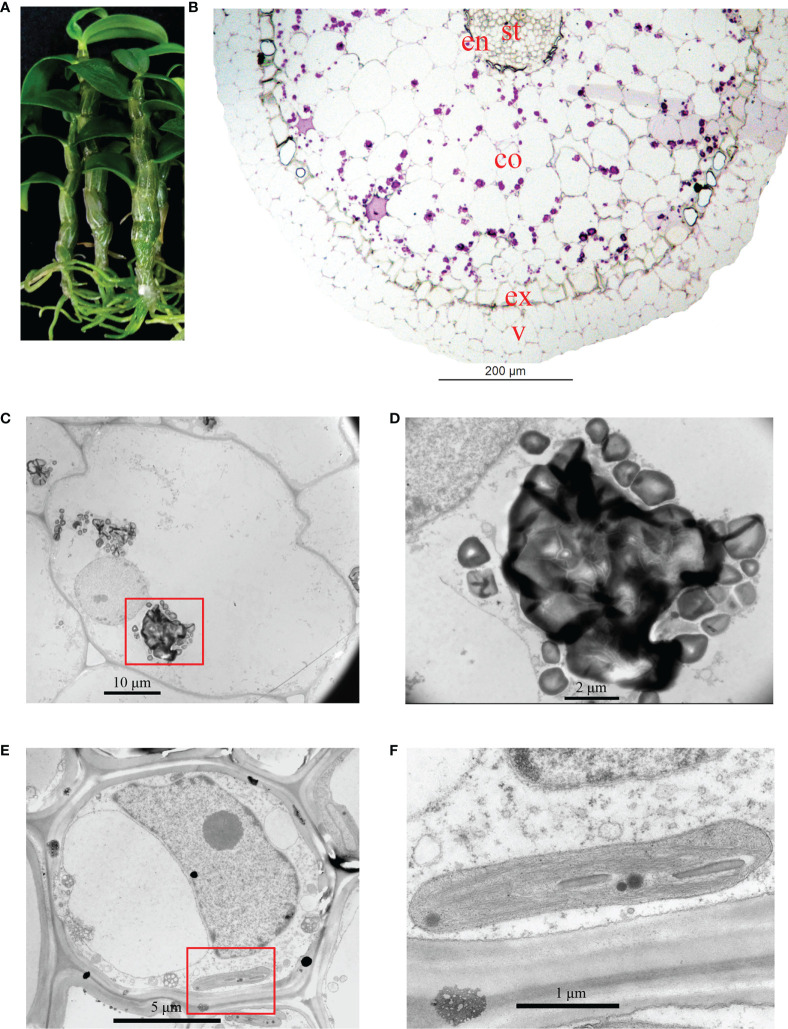
Analysis of the structure of *in vitro Dendrobium officinale* plantlet roots. **(A)** The roots of plantlets are green (chlorophyllous). **(B)** Overview of the anatomical traits of roots in a transverse section. Root tissue was stained by PAS. St, stele; en, endodermis; co, cortex; ex, exodermis; v, velamen. **(C)** Transmission electron microscopic observations of cortex cell morphology. **(D)** Enlarged view of starch granules from **(C)**. **(E)** Morphology of a stele cell. **(F)** Enlarged view of a chloroplast from **(E)**.

The roots of *D*. *officinale* plantlets were green and possessed meristematic and elongation zones (**Figure 1A**). The differentiation zone could not clearly or easily be distinguished from the meristematic and elongation zones because root hairs were absent (**Figure 1A**). To better understand the anatomical traits of *D*. *officinale* plantlet roots, we performed histological analysis to investigate the microstructure in transverse sections. The root consists of a velamen, cortex and stele, the largest proportion consisting of the cortex and a smaller stele (**Figure 1B**). The cortex is composed of an epidermis, an exodermis and a mass of cortical parenchyma tissue (**Figure 1B**). Starch granules (purple spots) were observed in the cortical parenchyma tissue but were absent in the stele and velamen (**Figure 1B**). Transmission electron microscopic observations revealed irregularly shaped starch granules that were easy to distinguish (**Figures 1C, D**). The stele cell contained a large (about 10 µm) nucleus and nucleolus, as well as small chloroplasts (**Figure 1E**). The thylakoids were stacked, similar to grana in leaves (**Figure 1F**). These observations demonstrate that *D*. *officinale* plantlet roots contain chloroplasts in their stele.

### Salinity induces multiple cellular events in roots of *D*. *officinale* plantlets

**Figure 2:**
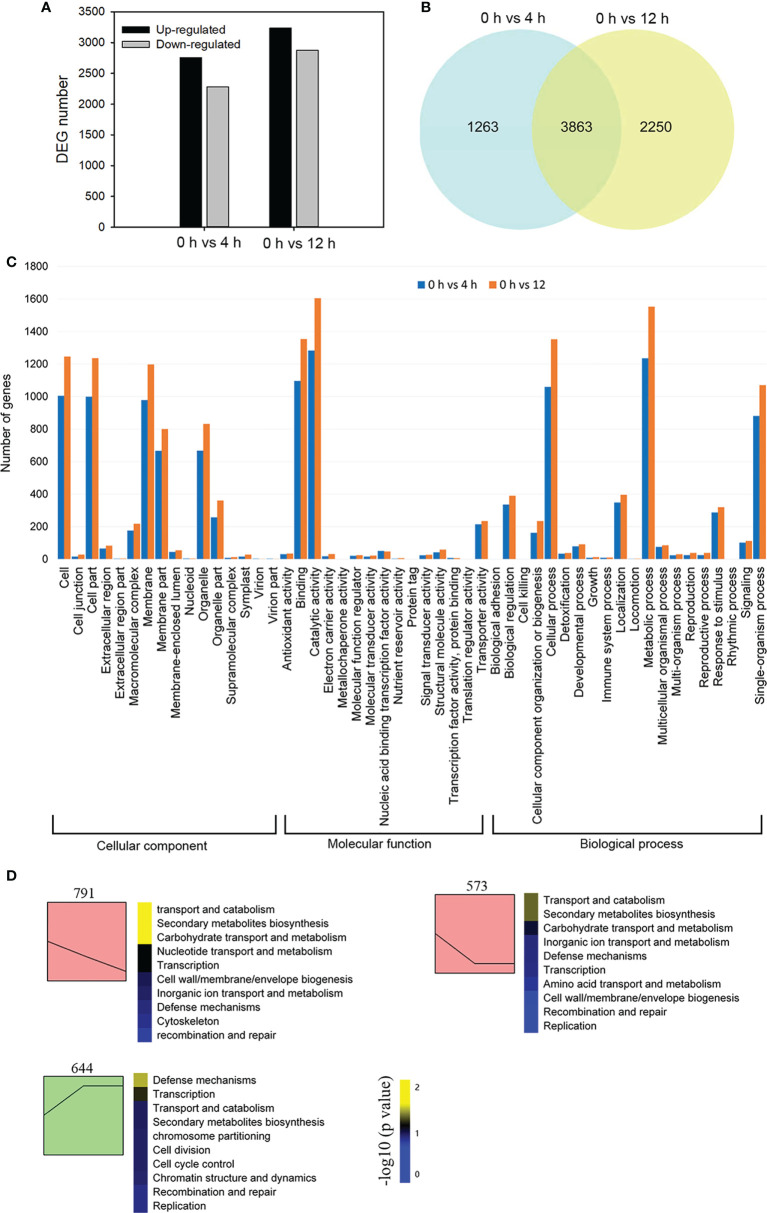
Transcriptome sequencing analysis of *Dendrobium officinale* plantlet roots after salt treatment. **(A)** Analysis of differentially expressed genes (DEGs) in response to salt tress. DEGs were generated by 0 h *vs* 4 h and 0 h *vs* 12 h comparisons. **(B)** Venn diagram showing shared DEGs from the 4 h and 12 h comparison after salt stress. **(C)** Gene ontology analysis of DEGs. **(D)** All DEGs were subjected to STEM clustering analysis.

In order to reveal global transcriptional dynamics in the roots of *D*. *officinale in vitro* plantlets exposed to salinity stress, we performed a time course transcriptomic analysis at 0, 4 and 12 h after exposure to 250 mM NaCl. In this study, total clean bases of each library generated from sequencing amounted to more than 6 Gb (**Supplementary Table 2**). The percentage of bases of each library having a quality score of 30 or higher exceeded 93% (**Supplementary Table 2**). We verified the expression patterns of 12 selected genes by qRT-PCR (**Supplementary Figure 1**). These were in agreement with the changes in fragments per kilobase per million (FPKM) values, supporting the reliability of our RNA-seq data. A total of 7376 DEGs were identified in the 0 h *vs* 4 h and 0 h *vs* 12 h comparisons. DEGs from the 0 h *vs* 4 h comparison consisted of 2282 down-regulated genes and 2844 up-regulated genes, while the number of DEGs from the 0 h *vs* 12 h comparison consisted of 2877 down-regulated genes and 3236 up-regulated genes (**Figure 2A**). Among all DEGs, 3863 genes showed a differential expression pattern at both time points: 2155 genes were up-regulated and 1702 genes were down-regulated (**Figure 2B**). We first performed a GO classification analysis of all the DEGs: 2538 and 3098 DEGs were assigned to three major GO categories (biological processes, cellular components, and molecular functions) in the 0 h *vs* 4 h and 0 h *vs* 12 h comparisons, respectively (**Figure 2C**). In the biological processes category, ‘metabolic process’ contained the greatest number of DEGs in both comparisons (**Figure 2C**). In the molecular function category, most DEGs were assigned to ‘catalytic activity’ in both comparisons (**Figure 2C**). To decipher the general trends of gene expression profiles, all DEGs were subjected to STEM clustering analysis (**Figure 2D**). These results suggest that salinity stress triggers a series of biological processes and molecular functions that mainly affect metabolism and defense in *D*. *officinale* roots.

### Auxin, cytokinin, ABA, ethylene and JA are involved in salinity response

**Figure 3:**
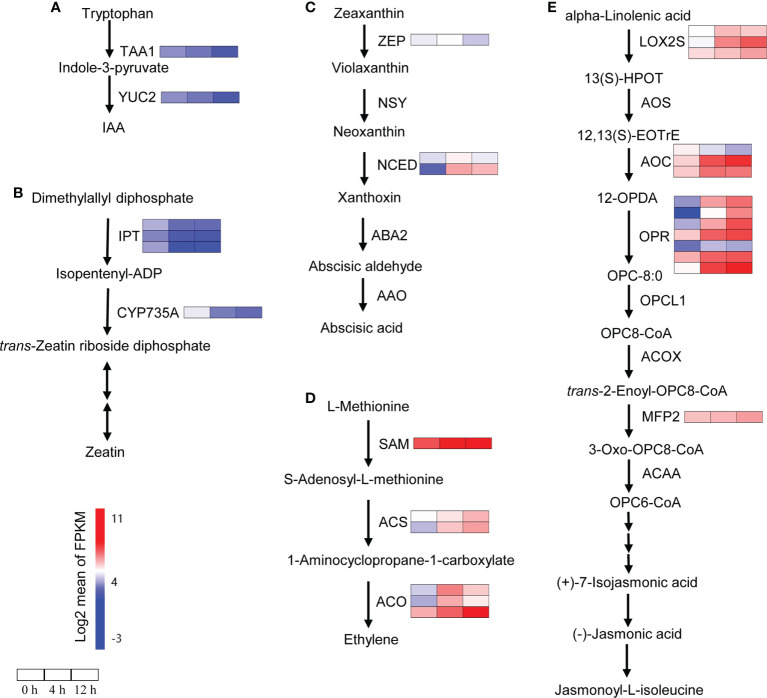
Changes in the expression of biosynthetic genes encoding phytohormones after salt stress. **(A)** Expression analysis of IAA biosynthetic genes. IAA, indole-3-acetic acid; TAA, tryptophan aminotransferase of Arabidopsis; YUC, indole-3-pyruvate monooxygenase 2. **(B)** Expression analysis of zeatin biosynthetic genes. IPT, isopentenyltransferase; CYP735A, cytokinin *trans*-hydroxylase. **(C)** Expression analysis of ABA biosynthetic genes. ABA, abscisic acid; ZEP, zeaxanthin epoxidase; NSY, neoxanthin synthase; NCED, 9-*cis*-epoxycarotenoid dioxygenase; ABA2, xanthoxin dehydrogenase; AAO, abscisic-aldehyde oxidase. **(D)** Expression analysis of ethylene biosynthetic genes. SAM, S-adenosylmethionine synthetase; ACS, 1-aminocyclopropane-1-carboxylate synthase; ACO, aminocyclopropanecarboxylate oxidase. **(E)** Expression analysis of JA biosynthetic genes. LOX2S, lipoxygenase; AOS, hydroperoxide dehydratase; AOC, allene oxide cyclase; OPR, 12-oxophytodienoic acid reductase; OPCL1, OPC-8:0 CoA ligase 1; ACX, acyl-CoA oxidase; MFP2, enoyl-CoA hydratase/3-hydroxyacyl-CoA dehydrogenase 2; ACAA, acetyl-CoA acyltransferase. Genes that changed significantly at least one time point after salt treatment are shown. Red indicates high expression and blue indicates low expression.

To analyze the relationship between plant hormones and salinity response in *D*. *officinale* roots, we investigated the genes related to the biosynthesis of plant hormones under salinity stress using RNA-seq data. The indole-3-acetic acid (IAA) biosynthetic pathway genes *TAA1* and *YUC2* showed a down-regulated expression pattern after salinity stress (**Figure 3A**). The genes involved in zeatin biosynthesis showed a similar expression pattern as the IAA biosynthetic pathway genes. For example, the expression of three *IPT* genes (encoding ADP/ATP‐dependent enzymes, isopentenyltransferases) and one cytokinin *trans*-hydroxylase gene *CYP735A* was repressed after salinity stress (**Figure 3B**). As expected, most of the genes involved in ABA, ethylene and JA biosynthesis were induced by salinity stress (**Figures 3C-E**). The *NCED* genes, which encode a key enzyme (9-*cis*-epoxycarotenoid dioxygenase) for ABA biosynthesis, were induced by salinity stress. The expression of one *NCED* gene increased at 4 h but dropped to its initial level at 24 h; another *NCED* gene was detected strongly at both 4 h and 12 h (more than 85-fold increase in expression) (**Figure 3C**). The DEGs related to ethylene biosynthesis increased at both time points after salinity treatment (**Figure 3D**). In total, we found that among the 14 DEGs related to JA biosynthesis, 13 were up-regulated at least at one time point while only one gene *AOC* was down-regulated at both time points (**Figure 3E**).

**Figure 4:**
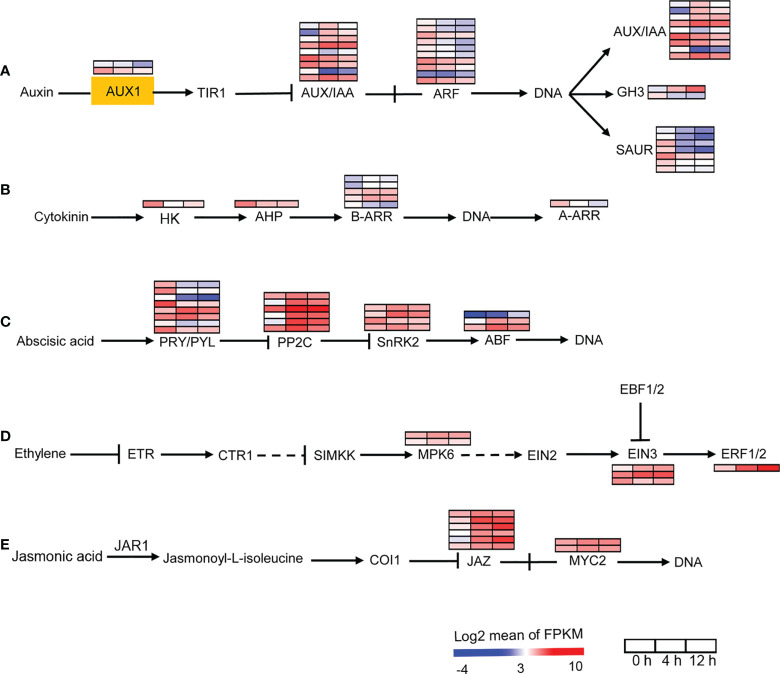
Changes in the expression of plant hormone signal transduction pathway under salt treatment. **(A)** Expression analysis of auxin signal transduction pathway genes. AUX1, auxin influx carrier 1; TIR1, transport inhibitor response 1; Aux/IAA, auxin/indole acetic acid protein; ARF, auxin response factor; GH3, Gretchen Hagen; SAUR, small auxin-up RNAs. **(B)** Expression analysis of cytokinin signal transduction pathway genes. HK, histidine kinase; AHP, histidine-containing phosphotransfer protein; A-ARR, type‐A Arabidopsis response regulator; B-ARR, type‐B Arabidopsis response regulator. **(C)** Expression analysis of ABA signal transduction pathway genes. PRY/PRL, abscisic acid receptor PYR/PYL family; PPC2C, protein phosphatase-2C; SnRK2, SNF1‐related protein kinase 2, ABF, ABA responsive element binding factor. **(D)** Expression analysis of ethylene signal transduction pathway genes. ETR, ethylene receptor; CTR1, serine/threonine-protein kinase 1; SIMKK, mitogen-activated protein kinase kinase; MPK6, mitogen-activated protein kinase 6; EIN2/3, ethylene-insensitive protein 2/3; EBF1/2, EIN3‐binding F‐box protein 1/2; ERF1/2, ethylene-responsive transcription factor 1/2. **(E)** Expression analysis of JA signal transduction pathway genes. JAR1, jasmonic acid-amino synthetase; COI1, coronatine-insensitive protein 1, MYC2, transcription factor MYC2. The pathways were redraw based on ‘Plant hormone signal transduction’ in the KEGG database (https://www.kegg.jp/). Genes that changed significantly in at least one time point after salt treatment are shown.

We then analyzed the auxin, cytokinin, ABA, ethylene and JA signal transduction pathway genes. AUX1 is a transmembrane amino acid transporter family protein that is involved in an early step of auxin signaling. Two *AUX1* homologs found in *D*. *officinale* were down-regulated after salinity treatment (**Figure 4A**). Our data showed the down-regulation of auxin responsive factors (ARFs) in response to salt stress (**Figure 4A**). In addition, auxin-responsive genes coding for proteins such as auxin/indole acetic acid (AUX/IAA), GH3, and small auxin-up RNA (SAUR) were differentially expressed after salinity stress, suggesting the importance of auxin in the salt stress response. For the cytokinin signal transduction pathway, signal transduction occurs *via* phosphotransfer between the sensor kinase and the receiver domain of the response regulator (Kieber and Schaller, 2018). In the cytokinin signal transduction pathway, *HK2*/*3*, which encodes a histidine kinase and *AHP*, which encodes a histidine-containing phosphotransfer factor, were down-regulated after salinity stress at both time points (**Figure 4B**). There are two types of response regulators (B-ARRs and A-ARRs) involved in cytokinin signaling (Kieber and Schaller, 2018). Type-B ARRs directly activate type-A ARRs in response to cytokinin (Kieber and Schaller, 2018). Four *B*-*ARR* genes were up-regulated and one was down-regulated after salt stress (**Figure 4B**). One *A*-*ARR* gene was repressed by salinity stress (**Figure 4B**). In addition, our transcriptomic analysis showed that more that 50% of ABA-, ethylene- and JA-responsive genes were up-regulated after salt stress (**Figures 4C-E**). These results indicate that the biosynthesis and signal transduction of auxin and cytokinin were repressed while the biosynthesis and signal transduction of ABA, ethylene and JA were activated in response to salinity stress, suggesting that auxin, cytokinin, ABA, ethylene and JA may be required for the response of *D*. *officinale* roots to salinity.

### The SOS pathway was activated in response to salinity

**Figure 5:**
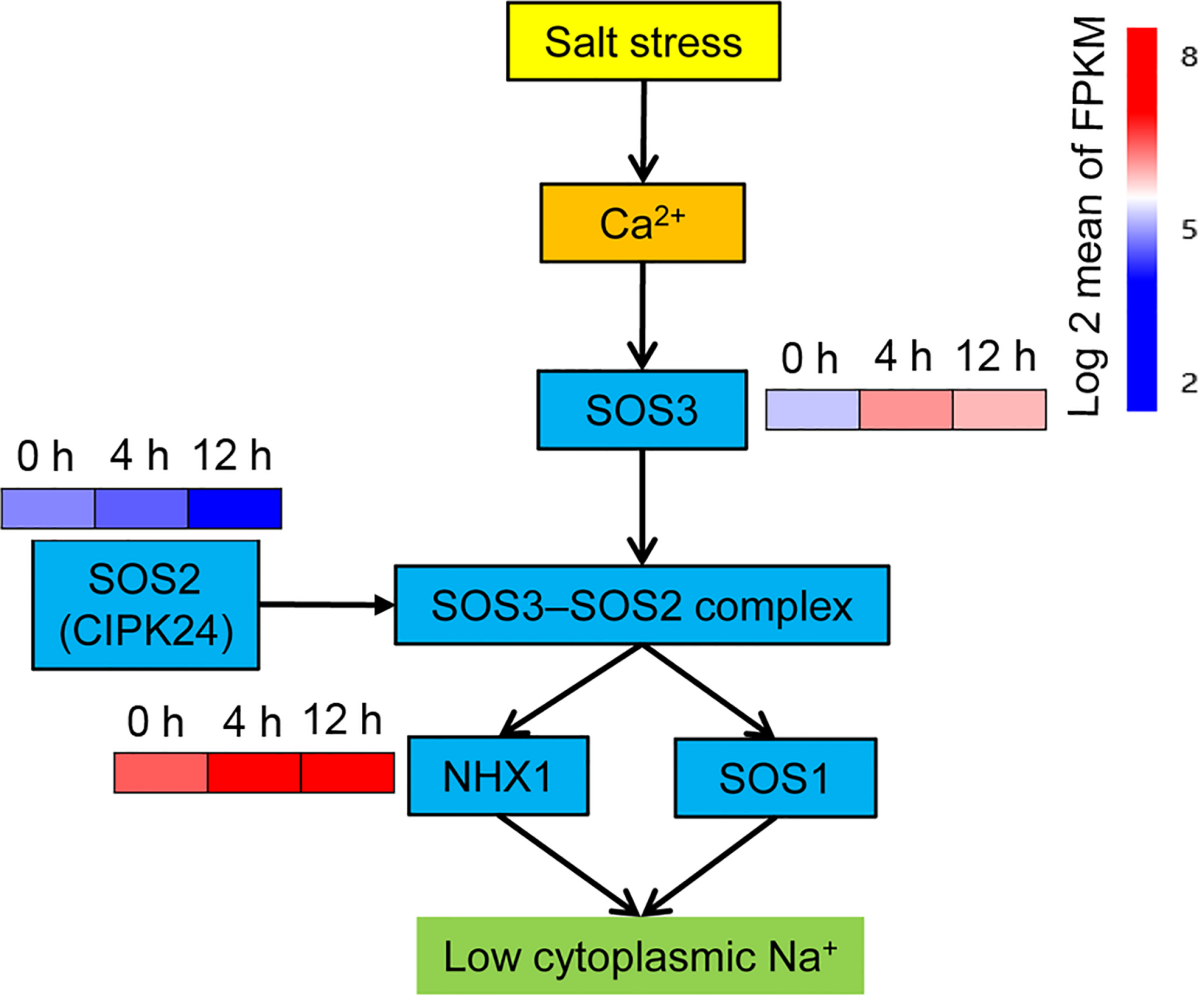
Expression analysis of SOS pathway genes. SOS, salt overly sensitive. The pathways were redrawn according to Tuteja (2007). Genes that changed significantly in at least one time point after salt treatment are shown.

In addition to the plant hormone signaling transduction pathway, another plant signaling pathway – the calcium signaling pathway – is also involved in the salt stress response. The SOS signaling network is activated by Ca^2+^ signaling (Kaleem et al., 2018). Three genes *Salt Overly Sensitive 1*-*3* (*SOS1*-*3*) are require for salt tolerance in plants (Ji et al., 2013). SOS3, which is a calcineurin B‐like protein that serves as a Ca^2+^‐binding protein, transduces the signal downstream by sensing changes in Ca^2+^ concentration in the cytoplasm (Tuteja, 2007). As expected, the *SOS3* gene was rapidly up-regulated at 4 h, but decreased at 12 h relative to 4 h (**Figure 5**). *SOS2* (also known as *AtCIPK24*) encodes a CBL-interacting serine/threonine-protein kinase (CIPK), which interacts with the SOS3 protein to form the SOS3–SOS2 protein kinase complex (Tuteja, 2007). Only one CIPK DEG, annotated as *CIPK24*, was found in this study, and its expression was repressed after salinity stress treatment (**Figure 5**). The *SOS1* gene encodes a Na^+^/H^+^ antiporter that results in a low concentration of cytoplasmic Na^+^ ions by enabling an efflux of excess Na^+^ ions across the plasma membrane. The vacuolar Na^+^/H^+^ exchanger 1 (NHX1) can reduce cytoplasmic Na^+^ by transferring cytoplasmic Na^+^ into vacuoles and maintaining osmotic balance in vacuoles (Apse et al., 1999; Bhaskaran and Savithramma, 2011). The SOS3–SOS2 protein kinase complex activates both SOS1 and NHX to trigger an Na^+^ exclusion response (Tuteja, 2007). No differential expression of the *SOS1* gene was observed in this study, while the expression of one gene encoding a vacuolar Na^+^/H^+^ exchanger was up-regulated after salt stress, with 2.4-fold and 2.2-fold greater expression than the control at 4 h and 12 h, respectively (**Figure 5**).

### Photosynthetic pigments, photosynthesis and carbon assimilation were impaired by salinity stress

**Figure 6:**
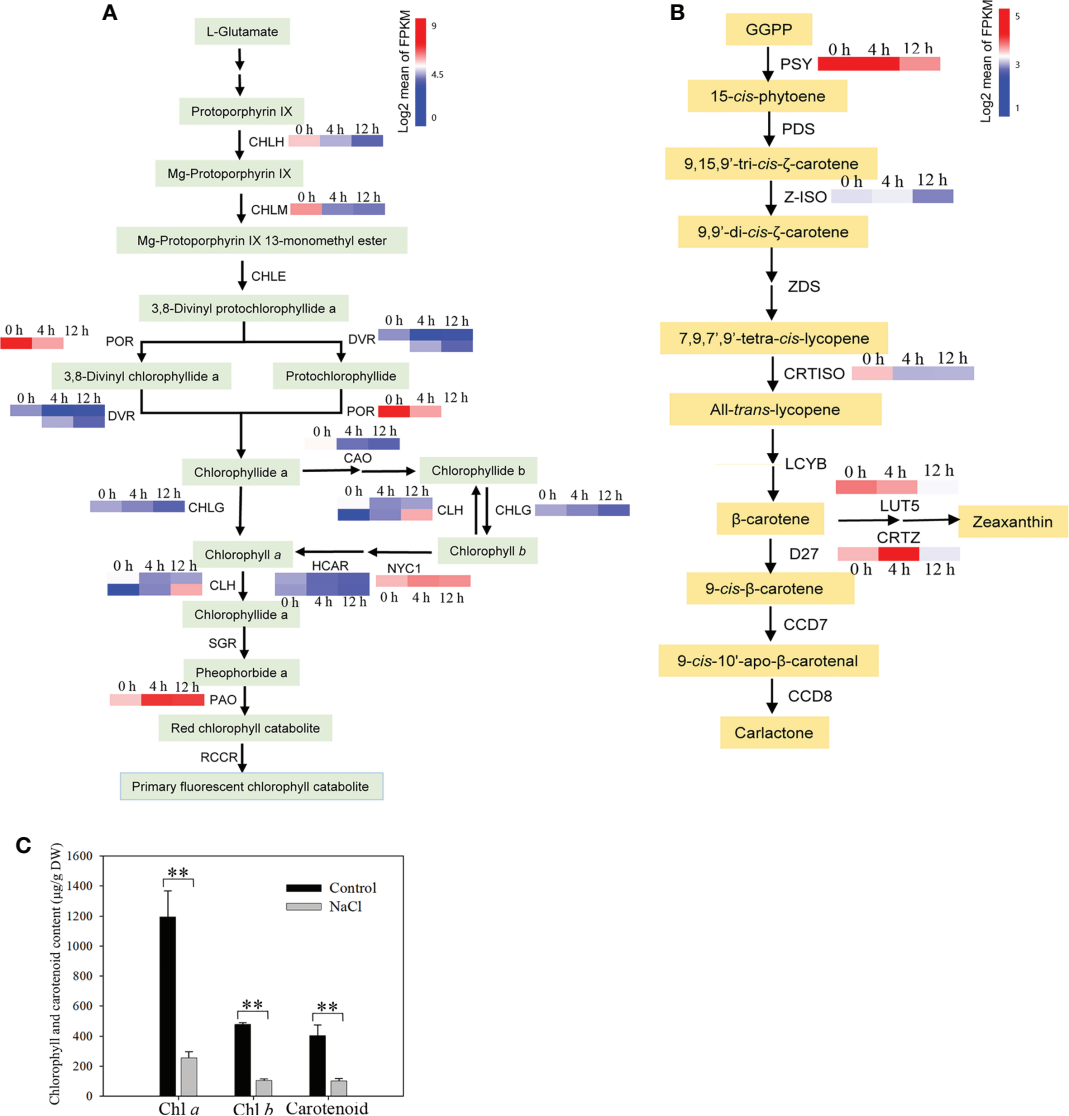
Analysis of the content of photosynthetic pigments and their metabolic pathway genes after salt treatment. **(A)** Analysis of expression of genes related to the Chl metabolic pathway. CHLH, magnesium chelatase subunit H; CHLM, magnesium-protoporphyrin *O*-methyltransferase; CHLE, magnesium-protoporphyrin IX monomethyl ester (oxidative) cyclase; POR, protochlorophyllide reductase; DVR, divinyl chlorophyllide *a* 8-vinyl-reductase; CHLG, chlorophyll/bacteriochlorophyll *a* synthase; CAO, chlorophyllide *a* oxygenase; CLH, chlorophyllase; NYC1, chlorophyll(ide) *b* reductase 1; HCAR, 7-hydroxymethyl chlorophyll *a* reductase; SGR, magnesium dechelatase; PAO, pheophorbide *a* oxygenase; RCCR, red chlorophyll catabolite reductase. **(B)** Changes in carotenoid biosynthetic pathway genes in response to salt stress. GGPP, geranylgeranyl diphosphate; PSY, 15-*cis*-phytoene synthase; PDS, 15-*cis*-phytoene desaturase; Z-ISO, ζ-carotene isomerase; ZDS, ζ-carotene desaturase; CRTISO, carotene *cis*-*trans* isomerase; LCYB, lycopene β-cyclase; LUT5, β-ring hydroxylase; CRTZ, β-carotene 3-hydroxylase; D27, β-carotene isomerase; CCD7, 9-*cis*-β-carotene 9’,10’-cleaving dioxygenase; CCD8, carlactone synthase/all-*trans*-10’-apo-β-carotenal 13,14-cleaving dioxygenase. The Chl metabolic pathway and carotenoid biosynthetic pathway were redrawn based on ‘porphyrin and chlorophyll metabolism’ and ‘carotenoid biosynthesis’ pathways in the KEGG database (https://www.kegg.jp/). Genes whose expression changed significantly in at least one time point after salt treatment are shown. **(C)** Content of chlorophyll (Chl) *a*, Chl *b* and carotenoids. Roots were harvested from plantlets after 250 mM NaCl treatment for two weeks. Bars indicate means ± standard deviation of three replicates. ** indicates significant differences at *P* < 0.01 according to the Dunnett test. DW, dry weight.

**Figure 7:**
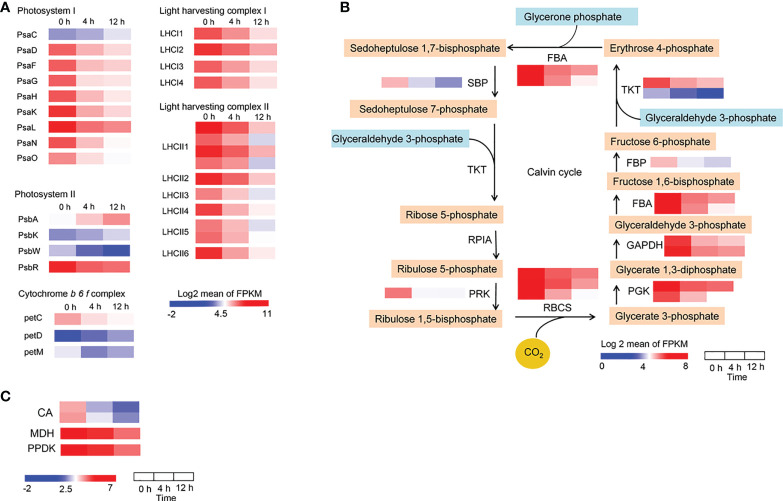
Changes in the expression of photosynthetic genes in response to salt treatment. **(A)** Expression pattern of genes encoding photosynthetic proteins, including the photosystem I complex, light harvesting complex I, photosystem II complex, light harvesting complex II, and cytochrome *b_6_f* complex. **(B)** Heatmaps of genes involved in the Calvin cycle. SBP, sedoheptulose-1,7-bisphosphatase; TKT, transketolase; RPIA, ribose 5-phosphate isomerase A; PRK, phosphoribulokinase; RBCS, ribulose-bisphosphate carboxylase small chain; PGK, phosphoglycerate kinase; GAPDH, glyceraldehyde 3-phosphate dehydrogenase; FBA, fructose-bisphosphate aldolase; FBP, fructose-1,6-bisphosphatase I **(C)** Heatmaps of genes involved in the CAM pathway. CA, carbonic anhydrase; MDH, malate dehydrogenase; PPDK, pyruvate, phosphate dikinase. Genes that changed significantly at least one time point after salt treatment are shown.

When salt concentration increases, this results in osmotic stress and the absorption of more Na^+^ and Cl^−^ by roots, which may negatively affect plant growth by decreasing photosynthetic efficiency (Deinlein et al., 2014). To reveal the impact of photosynthesis in *D*. *officinale* roots in response to salinity stress, we first analyzed photosynthesis-related biosynthetic genes and pigments. After examining the transcription profiles of Chl biosynthesis and catabolic genes, we found that all the Chl biosynthesis genes, except for one chlorophyll(ide) *b* reductase gene *NYC1* and one chlorophyllase gene *CLH2*, the expression of all other genes was down-regulated (**Figure 6A**). For example, the protochlorophyllide reductase gene *POR* was strongly expressed at 0 h with an average FPKM value > 750, but its expression dropped rapidly after salt stress treatment, with average FPKM values of 137 and 46 at 4 and 12 h, respectively (**Figure 6A**). In addition, the pheophorbide a oxygenase gene *PAO*, which encodes a key enzyme in the catabolism of Chl, was considerably up-regulated (with an average FPKM of about 90 at 0 h in contrast to an average FPKM value of > 500 at both 4 and 12 h) (**Figure 6A**). These results suggest that the decrease in Chl biosynthesis and reduced Chl degradation led to a decrease in Chl content in roots under salinity stress. In the carotenoid biosynthetic pathway, all of the identified carotenoid biosynthetic genes, except for *CRTISO2*, showed lowest expression levels at 12 h, but some of these genes were up-regulated at 4 h in response to salinity stress (**Figure 6B**). For example, the β-carotene 3-hydroxylase gene *CRTZ* had a mean FPKM value of 11 at 0 h, 57 at 4 h and 7 at 12 h (**Figure 6B**). However, Chl *a*, Chl *b* and total carotenoid content were not different between the control and salinity stress at 24 h (**Supplementary Figure 2**), but pigment content, especially Chl *a*, decreased significantly after two weeks’ exposure to salt stress (**Figure 6C**). These results indicate that photosynthetic pigments were reduced after salt stress, even a long time after exposure. We also isolated the DEGs that encode photosynthetic proteins, including those associated with the photosystem I complex, light harvesting complex I, photosystem II complex, light harvesting complex II, and cytochrome (Cyt) *b_6_f* complex, as well as DEGs related to carbon fixation. A small number of photosynthetic genes were up-regulated, such as *PsaC*, *PsbA*, *PsbK* and *petD*, while all of the remaining genes were down-regulated in response to salt stress (**Figure 7A**). In addition, the expression of genes in the Calvin cycle decreased (**Figure 7B**). For example, three *RBCS* genes, which encode ribulose bisphosphate carboxylases, were strongly expressed before salinity treatment, but were down-regulated more than 2.5-fold after salt stress (**Figure 7B**). *D*. *officinale* is regarded as a facultative crassulacean acid metabolism (CAM) species (Zhang et al., 2014). Hence, the genes encoding key enzymes for CAM photosynthesis were identified and analyzed. Two *CA* genes that encode carbonic anhydrase were down-regulated (**Figure 7C**). Other genes involved in CAM photosynthesis, such as the malate dehydrogenase gene *MDH* and pyruvate phosphate dikinase gene *PPDK*, were highly expressed in the control, but were suppressed rapidly after salinity treatment (**Figure 7C**). These results indicate that photosynthesis was considerably repressed by salinity stress and that the CAM pathway was not active at an early stage of salt stress in *D*. *officinale* roots.

### Global analysis of the amino acid metabolic pathway genes and composition of free amino acids

**Figure 8:**
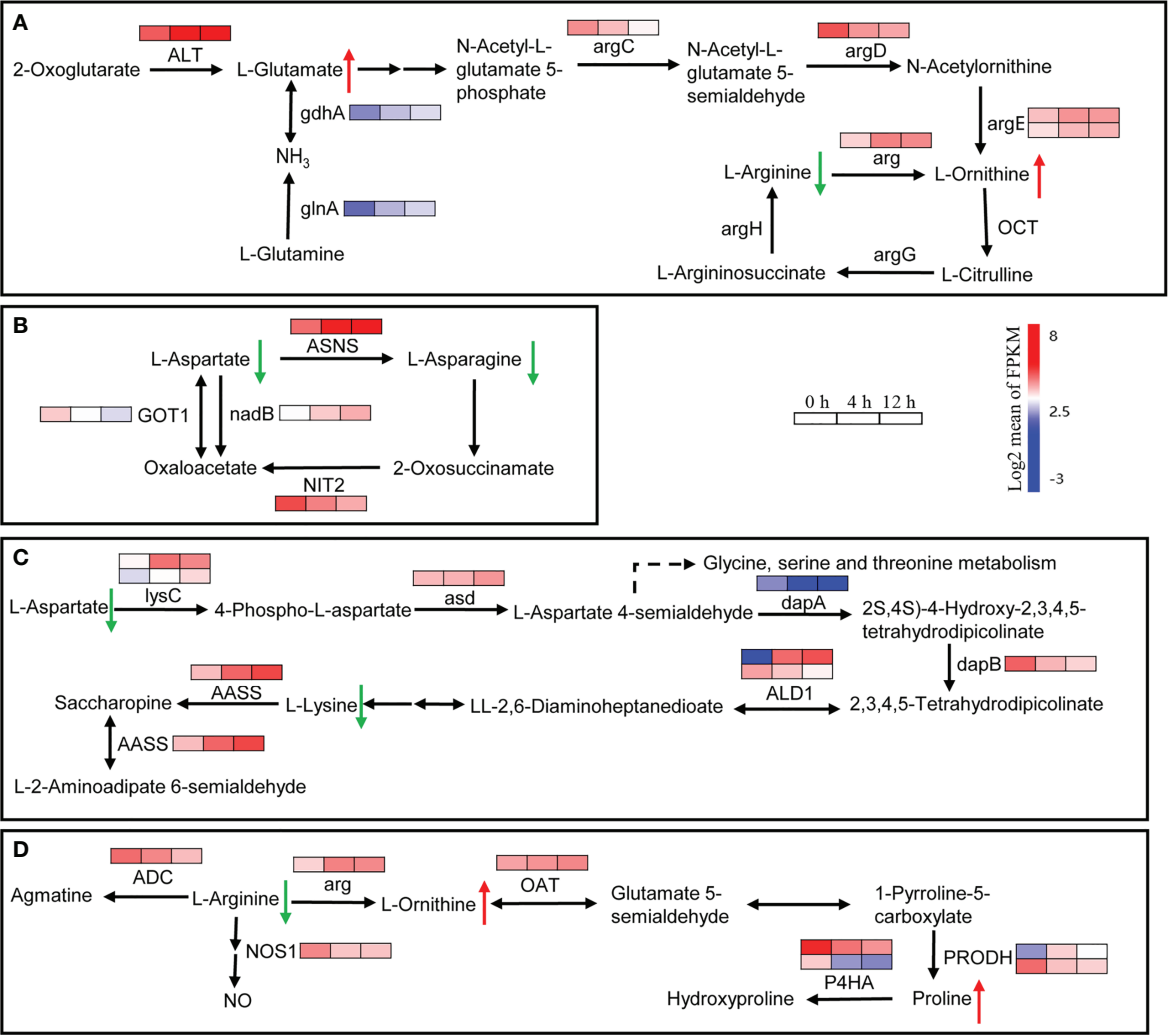
Expression heatmaps of genes involved in the selective amino acid metabolism pathway. **(A)** Changes in the expression of genes involved in glutamine, ornithine and arginine metabolism. ALT, alanine transaminase; argC, *N*-acetyl-γ-glutamyl-phosphate reductase; argD, acetylornithine aminotransferase; argE, acetylornithine deacetylase; argG, argininosuccinate synthase; arg, arginase; OCT, ornithine carbamoyltransferase; argH, argininosuccinate lyase; glnA, glutamine synthetase; gdhA, glutamate dehydrogenase. **(B)** Changes in the expression of genes involved in aspartate and asparagine metabolism. ASNS, asparagine synthase (glutamine-hydrolyzing); nadB, L-aspartate oxidase; GOT1, aspartate aminotransferase, cytoplasmic; NIT2, ω-amidase. **(C)** Changes in the expression of genes involved in aspartate and lysine metabolism. lysC, aspartate kinase; asd, aspartate-semialdehyde dehydrogenase; dapA, 4-hydroxy-tetrahydrodipicolinate synthase; dapB, 4-hydroxy-tetrahydrodipicolinate reductase; ALD1, LL-diaminopimelate aminotransferase; AASS, α-aminoadipic semialdehyde synthase. **(D)** Changes in the expression of genes involved in arginine, ornithine and proline metabolism. ADC, arginine decarboxylase; arg, arginase; OAT, ornithine aminotransferase; PRODH, proline dehydrogenase; P4HA, prolyl 4-hydroxylase; NOS1, nitric oxide synthase. Genes that changed significantly in at least one time point after salt treatment are shown.

In plants, amino acids serve as precursors for the synthesis of a wide range of biologically important compounds, but they also play a role in stress response. A total of 218 DEGs related to the amino acid metabolic pathway were found. They were clustered into two main groups: down-regulated DEGs after salt stress, and up-regulated DEGs at least one time point after treatment (**Supplementary Figure 3**). In the ‘Arginine biosynthesis’ pathway, the genes involved in glutamate and ornithine biosynthesis were up-regulated after salinity stress (**Figure 8A**). For example, *ALT*, *glnA* and *gdnA*, which are related to glutamate biosynthesis, increased at least 2-fold at 4 h and 3-fold at 12 h after salinity stress (**Figure 8A**). The *arg* gene, which encodes an arginase that converts arginine to urea and ornithine, was up-regulated about 3-fold after salinity stress (**Figure 8A**). The Asparagine synthetase gene *ASNS* and the L-aspartate oxidase gene *nadB* were up-regulated in response to salt stress (**Figure 8B**). In addition, two *lysC* genes that encode aspartate kinases catalyzing the synthesis of asparagine from aspartate were up-regulated more than 3-fold at 12 h after salinity stress (**Figure 8C**). In lysine metabolism, the gene *AASS*, which encodes α-aminoadipic semialdehyde synthase involved in lysine catabolism, was up-regulated (**Figure 8C**). In proline biosynthesis, one *PRODH* gene was up-regulated while another was down-regulated (**Figure 8D**). Moreover, two *P4HA* genes involved in proline catabolism were down-regulated (**Figure 8D**). The dry weight content of free amino acids in roots decreased about 2-fold between control and salinity stress, even two weeks after salinity treatment (**Table 1**). The content of all of the main free amino acids (asparaginate, *O-*phosphoethanolamine, arginine, lysine, and aspartate), except for *O*-phosphoethanolamine, declined after salinity stress (**Table 1**). The contents of proline and ornithine, which are involved in osmotic stress responses such as salt and drought stresses in plants (Hussein et al., 2019), were significantly up-regulated after salinity stress (**Table 1**). These results suggest that the biosynthesis of main free amino acids such as aspartate and arginine is blocked, while the biosynthesis of stress-related amino acids like proline and ornithine is promoted in response to salinity stress.

**Table 1:** Content of free amino acids (mg/100 mg DW) in *Dendrobium officinale* roots in control and salinity stress treatment.

Free amino acid	Control Mean ± SE	Salinity stress Mean ± SE	Regulation
*O*-Phospho-L-serine	0.0202 ± 0.0014	0.0207 ± 0.0018	
Taurine	0.0022 ± 0.0009	0.0110 ± 0.0010**	Up
*O*-Phosphoethanolamine	0.2150 ± 0.0176	0.2323 ± 0.0468	
L-Aspartic acid	0.0600 ± 0.0006	0.0453 ± 0.0030*	Down
L-Threonine	0.0216 ± 0.0004	0.0187 ± 0.0017	
L-Serine	0.0406 ± 0.0002	0.0740 ± 0.0045**	Up
L-Asparagine	3.8720 ± 0.0968	1.5855 ± 0.0665**	Down
L-Glutamic acid	0.0467 ± 0.0009	0.0610 ± 0.0050*	Up
D,L-α-Aminoadipic acid	0.0012 ± 0.0003	0.0012 ± 0.0002	
Glycine	0.0148 ± 0.0002	0.0127 ± 0.0013	
L-Alanine	0.0348 ± 0.0010	0.0430 ± 0.0040*	Up
L-Citrulline	0.0050 ± 0.0004	0.0103 ± 0.0060	
L-α-Amino-*n*-butanoic acid	0.0017 ± 0.0003	0.0020 ± 0.0006	
L-Cystine	0.0276 ± 0.0029	0.0615 ± 0.0029**	Up
L-Methionine	0.0027 ± 0.0006	0.0227 ± 0.0017**	Up
L-Isoleucine	0.0135 ± 0.0006	0.0148 ± 0.0015	
L-Leucine	0.0226 ± 0.0055	0.0200 ± 0.0029	
L-Tyrosine	0.0208 ± 0.0019	0.0252 ± 0.0037	
L-Phenylalanine	0.0175 ± 0.0009	0.0130 ± 0.0023	
β-Alanine	0.0010 ± 0.0000	0.0014 ± 0.0002	
D,L-β-Aminoisobutyric acid	0.0056 ± 0.0034	0.0008 ± 0.0002	
γ-Amino-*n*-butyric acid	0.0238 ± 0.0060	0.0468 ± 0.0093	
L-Histidine	0.0350 ± 0.0011	0.0323 ± 0.0024	
3-Methyl-L-histidine	0.0004 ± 0.0002	0.0005 ± 0.0003	
1-Methyl-L-histidine	ND	0.0004 ± 0.00024*	Up
L-Carnosine	0.0034 ± 0.0002	0.0017 ± 0.0005*	Down
L-Tryptophan	0.0022 ± 0.0007	0.0012 ± 0.0002	
Ornithine	0.0040 ± 0.0003	0.0062 ± 0.0005*	Up
L-Lysine	0.0805 ± 0.0016	0.0262 ± 0.0033**	Down
L-Arginine	0.9513 ± 0.0527	0.3297 ± 0.0182**	Down
L-Proline	0.0225 ± 0.0003	0.0240 ± 0.0003*	Up
Total	5.5923 ± 0.1356	2.7270 ± 0.2180**	Down

Mean values with an asterisk* in the same row are significantly different: * *P* < 0.05, ** *P* < 0.01, according to the Dunnet test. ND, not detected.

### Total flavonoids decreased in roots under salinity stress

**Figure 9:**
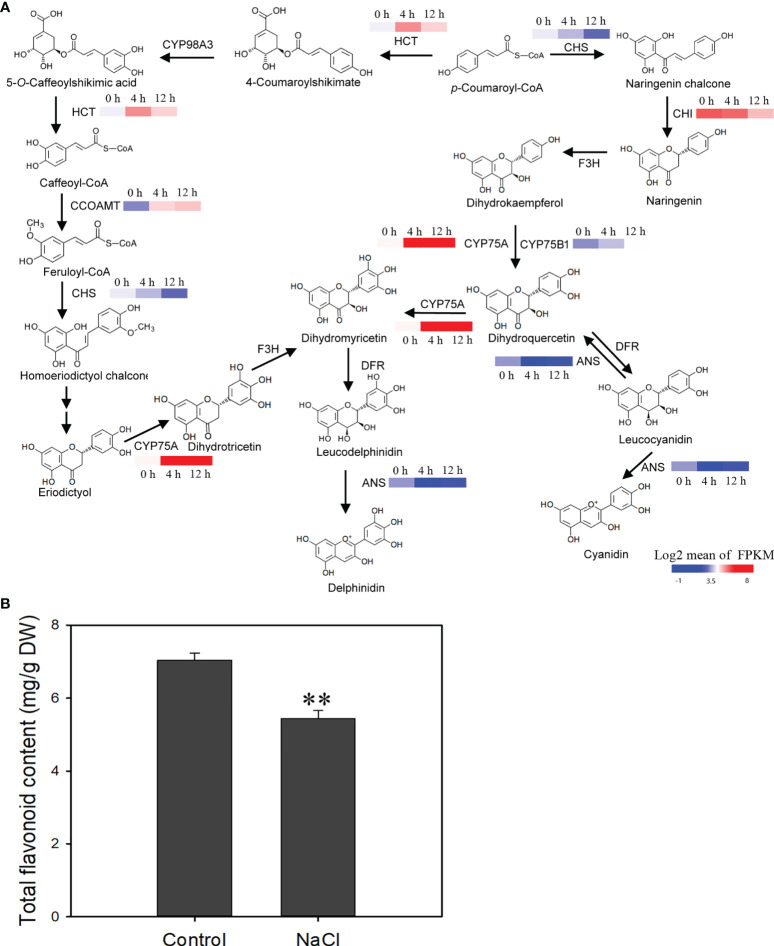
Changes in the expression of flavonoid biosynthetic genes and reduction of total flavonoids in response to salt treatment. **(A)** NaCl‐stress induced changes in the expression of flavonoid biosynthetic genes. CHS, chalcone synthase; HCT, shikimate *O*-hydroxycinnamoyltransferase; CYP98A, 5-O-(4-coumaroyl)-D-quinate 3’-monooxygenase; CCOAMT, caffeoyl-CoA *O*-methyltransferase; CHI, chalcone isomerase; F3H, naringenin 3-dioxygenase; CYP75A, flavonoid 3’,5’-hydroxylase; DFR, dihydroflavonol 4-reductase; ANS, anthocyanidin synthase. **(B)** Estimation of total flavonoid content after salt stress. The roots of plantlets were collected after 250 mM NaCl treatment for two weeks and used to measure total flavonoid content. Bars indicate means ± standard deviation of three replicates. ** indicates significant differences at *P* < 0.01 according to the Dunnett test. DW, dry weight. Genes that changed significantly in at least one time point after salt treatment are shown.

In the flavonoid metabolic pathway, the expression level of two genes coding for flavonoid 3′-hydroxylase (*CYP75A* and *CYP75B1*) increased after salinity stress, suggesting that the conversion of dihydrokaempferol to dihydroquercetin or dihydromyricetin was enhanced (**Figure 9A**). Flavonoid biosynthetic genes like *HTC* and *CCOAMT* were also affected after salinity stress and their expression was up-regulated (**Figure 9A**). However, expression of the genes coding for chalcone isomerase (*CHI*) and chalcone synthase (*CHS*), which are key enzyme genes in the synthetic pathway of flavonoids, declined after salinity stress (**Figure 9A**). Total flavonoid content decreased in roots after salinity stress (**Figure 9B**). These results suggest that the biosynthesis of flavonoids is suppressed by salinity stress in *D*. *officinale* roots.

### Global analysis of transcription factors and characterization of a *WRKY* gene in improvement of seed germination under salinity stress

**Figure 10:**
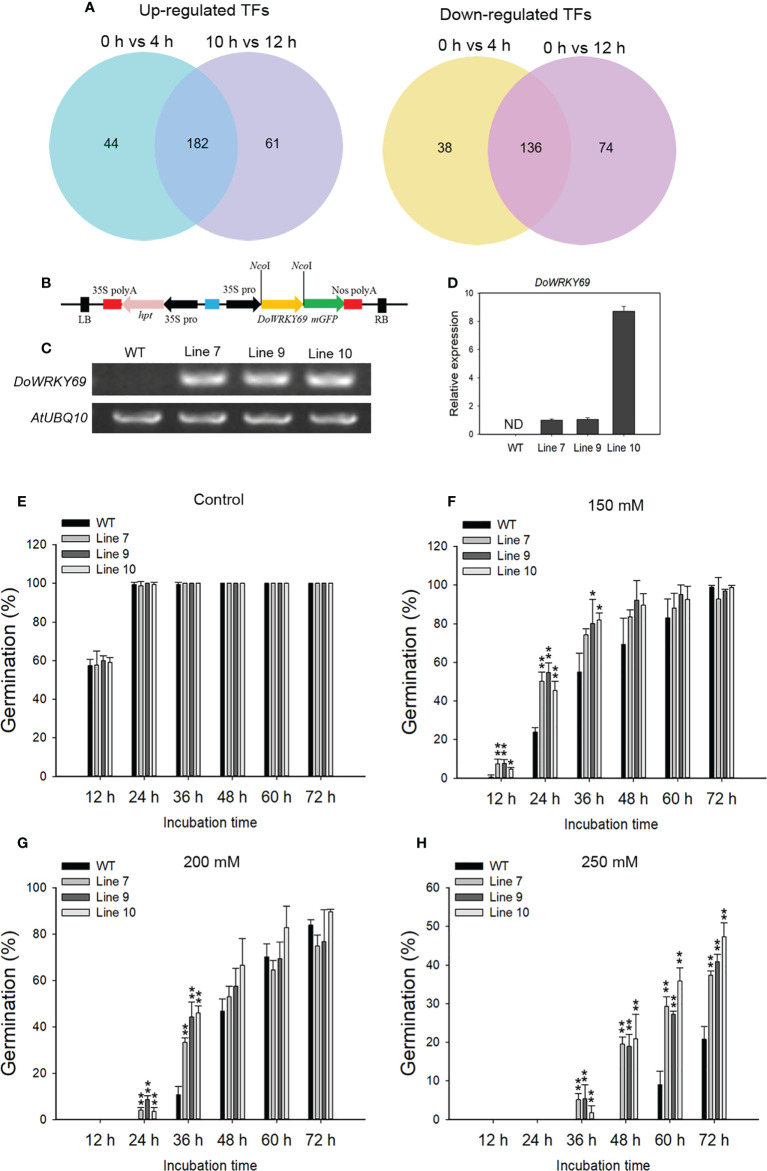
Identification of NaCl-induced TFs and characterization of one WRKY gene in the salt stress response. **(A)** Venn diagram of up-regulated TFs (left) and down-regulated TFs (right) from 0 h *vs* 4 h and 0 h *vs* 12 h comparisons. **(B)** The overexpression vector containing the *DoWRKY69* gene is shown. Analysis of the expression of the *DoWRKY69* gene in wild-type (WT) and *35S*::*DoWRKY69* lines by using RT-PCR **(C)** and qRT-PCR **(D)**. **(E)** Germination of WT and *35S*::*DoWRKY69* lines in the control (without NaCl), 150 mM NaCl **(F)**, 200 mM NaCl **(G)** and 250 mM NaCl **(H)**. Bars indicate means ± standard deviation of thee replicates. *, ** indicate significant differences at *P* < 0.05 and *P* < 0.01, respectively, according to the Dunnett test. About 60 seeds of each genotype were used.

TFs are widely involved in the regulation of metabolism and physiological processes, including stress response, in plants. A total of 226 and 243 TFs were up-regulated while 174 and 210 TFs were down-regulated at 4 and 12 h, respectively after salinity stress (**Figure 10A**). Among the down- or up-regulated genes at both time points, the largest number of down-regulated TFs was from the MYB family (22 genes) and the largest number of up-regulated TFs was from the APETALA2/ETHYLENE RESPONSE FACTOR (AP2/ERF) family (30 genes) (**Supplementary Figure 4**). Of note, all of the identified heat stress transcription factors were up-regulated at 4 h and 12 h. Only two WRKY genes were down-regulated while 17 WRKY genes were up-regulated at the detected time points after salt stress (**Supplementary Figure 4**).

To investigate the role of TFs in response to salinity stress, *DoWRKY69* with significantly up-regulated expression in WRKY family was selected and analyzed. *DoWRKY69* belongs to Group IIb (**Supplementary Figure 5**) and was up-regulated by about 4-fold at 4 h and by about 10-fold 12 h after salinity stress in roots (**Supplementary Figure 6**). The coding sequence (CDS) of *DoWRKY69* without a stop codon was cloned into pCAMBIA1302 at the *Nco*I site and is driven by the 35S promoter (**Figure 10B**). Ten independent transgenic lines for *DoWRKY69* were generated. Lines 7, 9 and 10 were used to analyze expression level and seed germination in response to three NaCl concentrations (150, 200, and 250 mM). The *DoWRKY69* gene was detected in all three transgenic lines but was not detected in WT plants (**Figures 10C, D**). In WT and transgenic lines, seeds germinated well and their germination percentage was nearly 100% in control medium 24 h after incubation (**Figure 10E**). However, the difference between WT and transgenic lines was obvious when the growth medium was supplemented with salt (**Figures 10F-H**). The germination rate of all transgenic lines was significantly higher than WT seeds within 24 h in response to 150 mM NaCl (**Figure 10F**). No WT seeds germinated within 24 h, while more than 3% of transgenic seeds of each transgenic line germinated with 24 h after exposure to 200 mM NaCl (**Figure 10G**). In addition, the germination of transgenic lines was significantly higher than WT seeds at 36 h after exposure to 200 mM NaCl (**Figure 10H**). Although no seeds germinated within 48 h in response to 250 mM NaCl, the germination of transgenic lines was significantly higher than WT seeds within 72 h after exposure to 250 mM NaCl (**Figure 10H**). These results suggest that *DoWRKY69* plays a positive role in salt stress tolerance in *A. thaliana*.

## Discussion

Salinity is one of the most severe environmental factors limiting the productivity of agricultural crops. It is necessary to explore salinity tolerance mechanisms to help improve salt tolerance of crops *via* genetic engineering. In this study, we investigated transcriptomic reprogramming in *D*. *officinale* plantlets at an early phase of salinity stress, and characterized the ability of an up-regulated gene *DoWRKY69* to improve *A. thaliana* seed germination under salt stress. The roots of *D*. *officinale* plantlets are photosynthetic. The biosynthesis and signal transduction of hormones, such as IAA and cytokinin, which are essential for plant growth and development, were repressed, while stress-responsive hormones such as ABA, JA and SOS pathway genes were up-regulated at an early phase in response to salt stress. In order to survive and adapt to salinity stress, physiological and metabolic adjustments were made through extensive transcriptomic reprogramming. Our results suggest that physiological and metabolic processes and molecular functions are reprogrammed in the photosynthetic roots of this orchid when plants are exposed to salinity stress.

Plant hormones are crucial signaling molecules and their signaling depends on their spatio-temporal distribution (Waadt, 2020). Phytohormones coordinate all aspects of plant growth and development, as well as stress responses (Shan et al., 2012). Plant hormones interact with five key plant neurotransmitters, including serotonin, melatonin, dopamine, acetylcholine and γ-aminobutyric acid, and participate in many physiological processes such as photosynthesis, oxidative stress and osmotic regulation (Raza et al., 2022b). Two groups of phytohormones, auxins and cytokinins, have been clearly demonstrated as the main regulators of plant development (Benjamins and Scheres, 2008; Werner and Schmülling, 2009; Schaller et al., 2015) and stress responses (Hare et al., 1997; Blakeslee et al., 2019). For example, the *YUC* gene, which encodes indole-3-pyruvate monooxygenase, plays an important role in auxin (IAA) biosynthesis (Zhao, 2012). Overexpression of the *YUC* gene exhibited a drought-resistant phenotype in *A. thaliana* (Lee et al., 2012), and conferred water-deficit tolerance in potato (Kim et al., 2013b). However, improvement of drought tolerance by overexpression of the *YUC6* gene was not due to an increase in auxin synthesis, but rather due to *YUC6*-processed thiol-reductase, which inhibited the generation of ROS (Cha et al., 2015). Only one *YUC* gene was identified in this study and its expression was down-regulated after salt stress, suggesting that the synthesis of IAA might decrease in *D*. *officinale* roots in response to salt stress (**Figure 3A**). Moreover, the IAA biosynthesis pathway gene *IAA1* and auxin signal-mediated genes (*AUX1* and *ARF*) were down-regulated after salt stress, suggesting a reduction in auxin signals in response to salinity stress. Cytokinin biosynthesis pathway genes (three *IPT* genes and *CYP735A*) and the expression of cytokinin signal transduction pathway genes (*HK*, *AHP* and *A*-*ARR*, as well as one *B*-*ARR* gene) declined in *D*. *officinale* roots after salt stress, indicating the inhibition of cytokinin signaling in roots after salt stress. Biosynthetic genes such as *CYP735A2* from *A. thaliana* and *IPT* (a key enzyme for cytokinin biosynthesis) from tomato were predominantly expressed in roots, suggesting that roots are a major site for cytokinin synthesis (Takei et al., 2004; Ghanem et al., 2011). The down-regulated expression of *IPT* genes and *CYP735A* in *D*. *officinale* roots indicates a decrease in cytokinin. Some studies have shown that cytokinin signaling plays a negative role in stress responses. For example, two histidine kinase genes (*AHK2* and *AHK3*) negatively controlled osmotic stress responses in *A. thaliana*, and their single or double mutants displayed strong tolerance to drought and salt stress (Tran et al., 2007). These results indicate that cytokinin was a negative regulator of stress and that repression of the cytokinin signal in *D*. *officinale* roots might help plants to cope with salt stress. Most of the genes related to the biosynthesis of ABA, ethylene and JA and their signal transduction pathways were up-regulated after salt stress in *D*. *officinale* roots (**Figures 3**, **4**). ABA, ethylene and JA signal transduction pathways after exposure to salt stress were described in greater detail in a fairly recent review (Kaleem et al., 2018). This indicates the conserved role of ABA, ethylene and JA in the response of plants to salt stress.

Photosynthesis is an essential process, converting solar energy into chemical energy. Salt stress plays a negative role in the photosynthesis of leaves (Sudhir and Murthy, 2004; Silva et al., 2011). Genes that encode the components of both the light and dark reactions of photosynthesis in the leaves of salt-treated soybean seedlings were slightly repressed or maintained at the early phase within 4 h, but were inhibited at a later phase (after 24 h) (Liu et al., 2019). Chloroplasts are important organelles central to plant photosynthesis and are affected by salt-induced toxicity, although different plant species and development stages display different degrees of resistance to salt stress (Suo et al., 2017; Hameed et al., 2021). In this study, an anatomical analysis demonstrated that *D*. *officinale* plantlet roots contained chloroplasts (**Figure 1**), suggesting that the roots were capable of photosynthesis. The genes involved in the biosynthesis of photosynthetic pigments, as well as the genes encoding photosynthetic components, were strongly repressed in roots of salt-treated *D*. *officinale* plantlets within 12 h. These results indicate that photosynthesis is inhibited in both roots and leaves in response to salt stress, while the influence of time in both organs is different. Photosynthetic pigments were not discernibly altered under short-term salt stress (within 24 h) in the roots of *D*. *officinale* plantlets, although the expression of biosynthetic genes dropped drastically within 12 h (**Figure 3**). It is possible that the photosynthetic pigments were not damaged by salt stress in the first 12 h of exposure.

A decrease in photosynthetic rates under stress (dehydration, salt, extended darkness) may lead to an insufficient supply of carbohydrates. Free amino acids, which are tightly linked to carbohydrate metabolism, can be used as alternative substrates for mitochondrial respiration or act as precursors for the biosynthesis of secondary metabolites or immune signaling metabolites (Hildebrandt et al., 2015; Chen et al., 2018; Hildebrandt, 2018). In this study, the biosynthesis of arginine was repressed and its breakdown was induced, leading to a decrease of arginine in *D*. *officinale* roots after exposure to salt stress, suggesting the arginine might be used as a precursor for the biosynthesis of other compounds. For example, polyamines, which are synthesized from arginine, are responsive to plant stresses (Alcázar et al., 2006; Groppa and Benavides, 2008). Auxin can be synthesized from tryptophan (Wang et al., 2015a) and ethylene is synthesized from methionine (Sauter et al., 2013). There were no differences in tryptophan content while the expression of auxin biosynthetic genes was repressed by salt stress (**Table 1** and **Figure 8A**). The increased expression of methionine and the deregulation of ethylene biosynthetic genes after salt stress (**Table 1** and **Figure 8D**) suggest that ethylene synthesis is related to the methionine cycle. In addition, the higher level of stress-related proline and organic acids has been observed in salt cress (*Eutrema salsugineum*) exposed to extreme salt stress (Li et al., 2022). In the roots of *D*. *officinale*, proline biosynthetic genes were induced by salt and their expression was correlated with the increase in proline content.

Flavonoids, a diverse group of bioactive polyphenolic compounds, are catalyzed by a series of enzymes. CHS is first rate-limiting enzyme in flavonoid biosynthesis and catalyzes the production of naringenin chalcone using *p*-coumaroyl-CoA as the starting substrate (Martens et al., 2010; Dao et al., 2011). Under salinity stress, *CHS* and *CHI* were down-regulated, and this might have led to a decrease of total flavonoid production in *D*. *officinale* roots. Flavonoids are effective antioxidants that are involved in the response mechanisms of plants under adverse environments, including biotic and abiotic stresses (Agati et al., 2012; Petrussa et al., 2013). Studies have shown that salinity stress can enhance total flavonoid concentration in plant leaves. For example, flavonoid biosynthesis pathway genes such as the phenylalanine ammonia lyase gene *PAL*, the chalcone synthase gene *CHS* and the flavonol synthase gene *FLS*, as well as total flavonoids, were up-regulated in the leaves of *Solanum nigrum* under salt stress (Ben Abdallah et al., 2016). In our previous study, flavonoid biosynthesis genes (such as *CHS*, *CHI* and *F3H*) were up-regulated and total flavonoid content was enhanced in *D*. *officinale* leaves under salt stress (Zhang et al., 2021). Flavonoids act as antioxidants by scavenging ROS in stressed plant leaves (Agati et al., 2012). However, the content of flavonoids decreased in *D*. *officinale* roots under salt stress, suggesting that the path or mechanism by which ROS is scavenged in the roots of *D*. *officinale* is different from that in leaves.

Transcriptional activation of functional genes involved in stress responses by TFs leads to the adjustment of specific metabolism of metabolites, which is one strategy to cope with stress in plants. Different studies have identified many TFs involved in regulating the response to salt stress (Kaleem et al., 2018). Most up-regulated TFs belong to AP2/ERF, MYB, NAC and WRKY families (Supplementary **Figure 3**). Accumulating evidence has shown that WRKY genes play positive or negative roles in the regulation of the salt stress response. Maize (*Zea mays*) *WRKY17* and *WRKY114* negatively regulate salt stress tolerance (Cai et al., 2017; Bo et al., 2020). *GhWRKY17* from cotton (*Gossypium hirsutum*) and *WRKY46* from *A. thaliana* contribute to salt stress tolerance (Yan et al., 2014; Ding et al., 2015). A WRKY gene *DoWRKY69* was up-regulated after salt stress treatment. Over-expression of *DoWRKY69* demonstrated its role in improving *A. thaliana* seed germination under salt stress. These results indicate that stress-related TFs can be induced by salt stress treatment and might be responsible for the stress response.

## Conclusion

**Figure 11:**
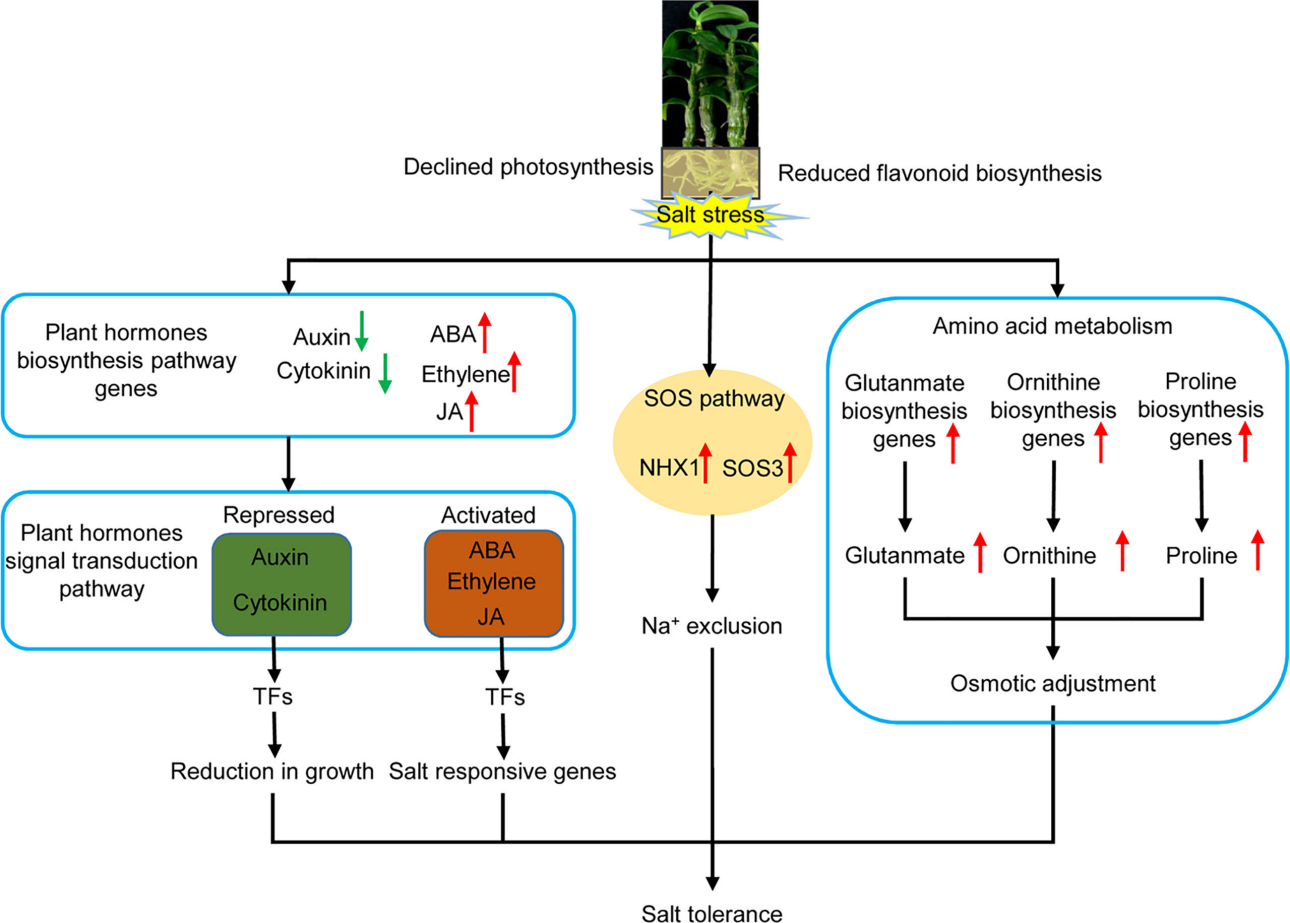
Schematic diagram of the response mechanism of *D*. *officinale* roots under salinity stress. Upward-facing red arrows represent activated/up-regulated targets. Downward-facing green arrows represent repressed/down-regulated genes.

During the salt stress response mechanism of *D. officinale in vitro* plantlets, the expression of genes involved in photosynthesis and flavonoid biosynthesis, as well as in the biosynthesis of auxin and cytokinin, declined, whereas the expression of genes coding for stress-related plant hormones (ABA, ethylene and JA), the signal transduction pathway, the SOS pathway, and the biosynthesis of amino acids related to osmotic adjustment, were activated (**Figure 11**). This indicates that *D*. *officinale* adapted to salt stress by reducing growth, the accumulation of compatible solutes, and increasing the exclusion of excess Na^+^ in roots (**Figure 11**). Our findings illustrate a response mechanism of the roots of a facultative CAM plant to salinity stress. Our study also provides a large number of candidate stress-responsive genes that could be used to develop and strengthen economically important crops using biotechnological approaches, allowing them to cope with salt stress.

## Data availability statement

The original contributions presented in the study are publicly available. This data can be found here: PRJNA715099

## Author contributions

CH and MZ supervised the project. MZ and CH conceived the research and designed the experiments. RD performed histological analysis. MZ, CH, NL, XL, YY, JT and JD collectively interpreted the results and wrote all drafts of the manuscript. All authors contributed to the article and approved the submitted version.

## Funding

This research was funded by the National Natural Science Foundation of China (32071819 and 31800204), the Youth Science and Technology Talent Growth Project of Education Department of Guizhou Province of China (No. [2022]099), the Science and Technology Department Foundation of Guizhou Province of China (No. [2020]1Y120), and the Project of High-Level Talents Introduction in Qiannan Normal University for Nationalities (2021qnsyrc06).

## Acknowledgments

We thank Dr. Minglei Zhao (South China Agricultural University) for helpful suggestions and Jian Liu (South China Botanical Garden, Chinese Academy of Sciences) for analyzing free amino acid content.

## Conflict of interest

The authors declare that the research was conducted in the absence of any commercial or financial relationships that could be construed as a potential conflict of interest.

## Publisher’s note

All claims expressed in this article are solely those of the authors and do not necessarily represent those of their affiliated organizations, or those of the publisher, the editors and the reviewers. Any product that may be evaluated in this article, or claim that may be made by its manufacturer, is not guaranteed or endorsed by the publisher.
