# Supplementary material for: Physiological and transcriptomic analysis uncovers salinity stress mechanisms in a facultative crassulacean acid metabolism plant Dendrobium officinale
Source: Front Plant Sci. 2022 Oct 6;13:1028245. doi: 10.3389/fpls.2022.1028245 (PMC9582936; doi:10.3389/fpls.2022.1028245)
Supplement: Supplementary file 1 [file DataSheet_1.docx]

**Physiological and Transcriptomic Analysis Uncovers Salinity Stress Mechanisms in a Facultative Crassulacean Acid Metabolism Plant *Dendrobium officinale***

Mingze Zhang^1,2^, Nan Liu^3^, Jaime A. Teixeira da Silva^4^, Xuncheng Liu^2^, Rufang Deng^5^, Yuxian Yao^1^, Jun Duan^2,6^, Chunmei He^2^*

^1^The Department of Life Science and Agriculture, Qiannan Normal University for Nationalities, Duyun, China

^2^Key Laboratory of South China Agricultural Plant Molecular Analysis and Genetic Improvement, Provincial Key Laboratory of Applied Botany, South China Botanical Garden, Chinese Academy of Sciences, Guangzhou, China

^3^Key Laboratory of Vegetation Restoration and Management of Degraded Ecosystems, South China Botanical Garden, Chinese Academy of Sciences, Guangzhou, China

^4^Independent researcher, Kagawa-ken, Japan

^5^Opening Public Laboratory, Chinese Academy of Sciences, Guangzhou, China

^6^Center of Economic Botany, Core Botanical Gardens, Chinese Academy of Sciences, Guangzhou, China

*Corresponding Author.


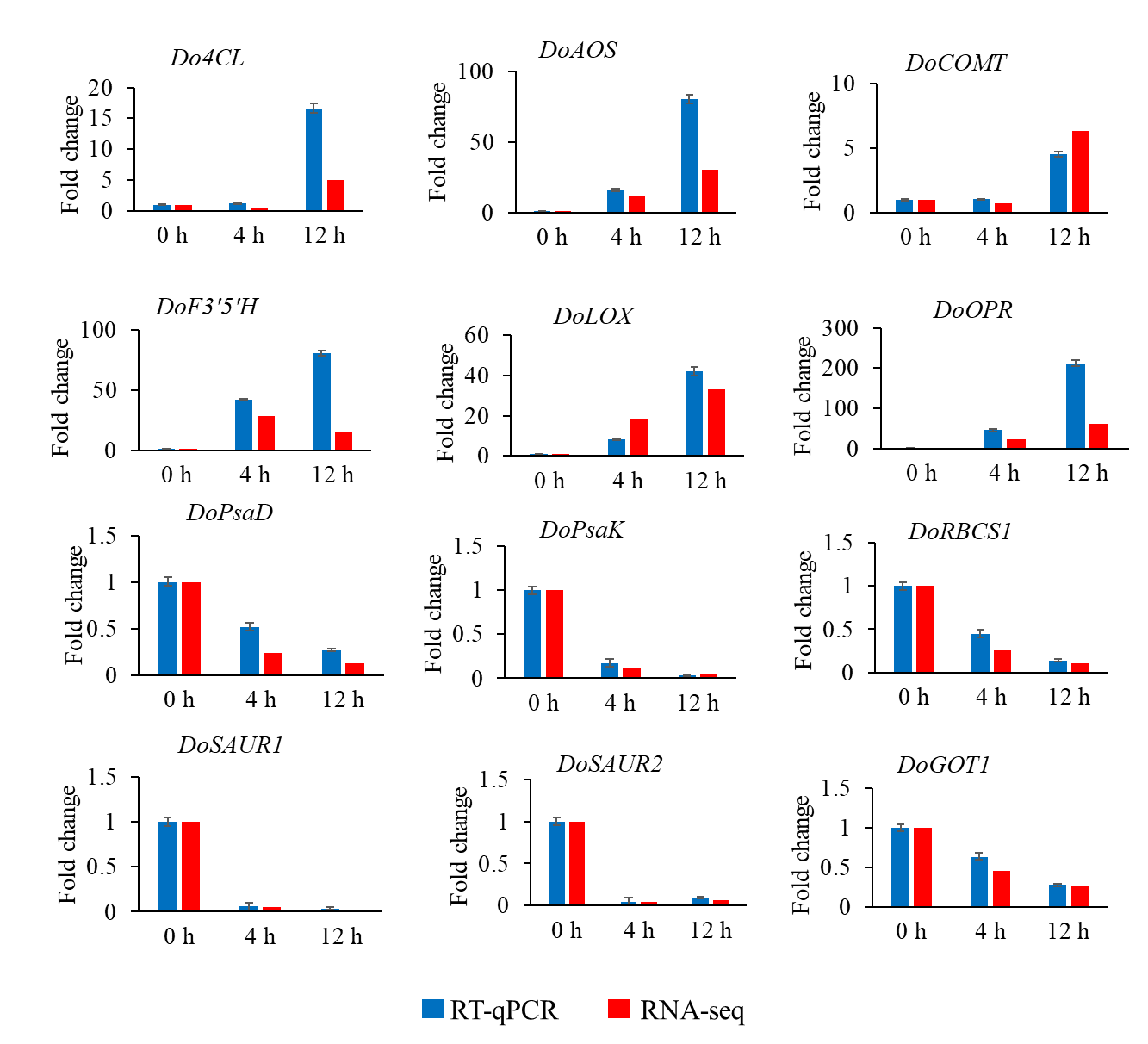


Supplementary Figure 1 Real-time qRT-PCR analyses to validate gene expression profiles deduced from RNA-seq. The expression profiles of 12 selected genes, *Do4CL*, *DoAOS*, *DoCOMT*, *DoF3’5’H*, *DoLOX*, *DoOPR*, *DoPsaD*, *DoPsaK*, *DoRBCS1*, *DoSAUR1*, *DoSAUR2* and *DoGOT1*, were validated.


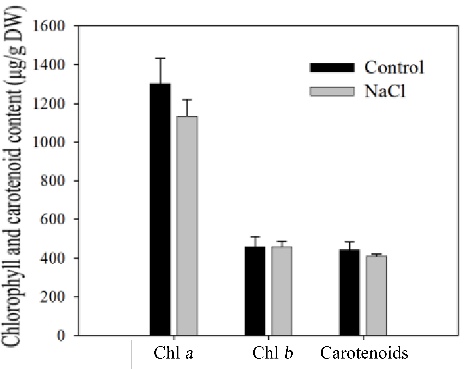


Supplementary Figure 2 Content of chlorophyll (Chl *a*, Chl *b*) and carotenoids. Roots were harvested from plantlets after 250 mM NaCl treatment at 24 h. Bars indicate means ± standard deviation of three replicates. DW, dry weight.


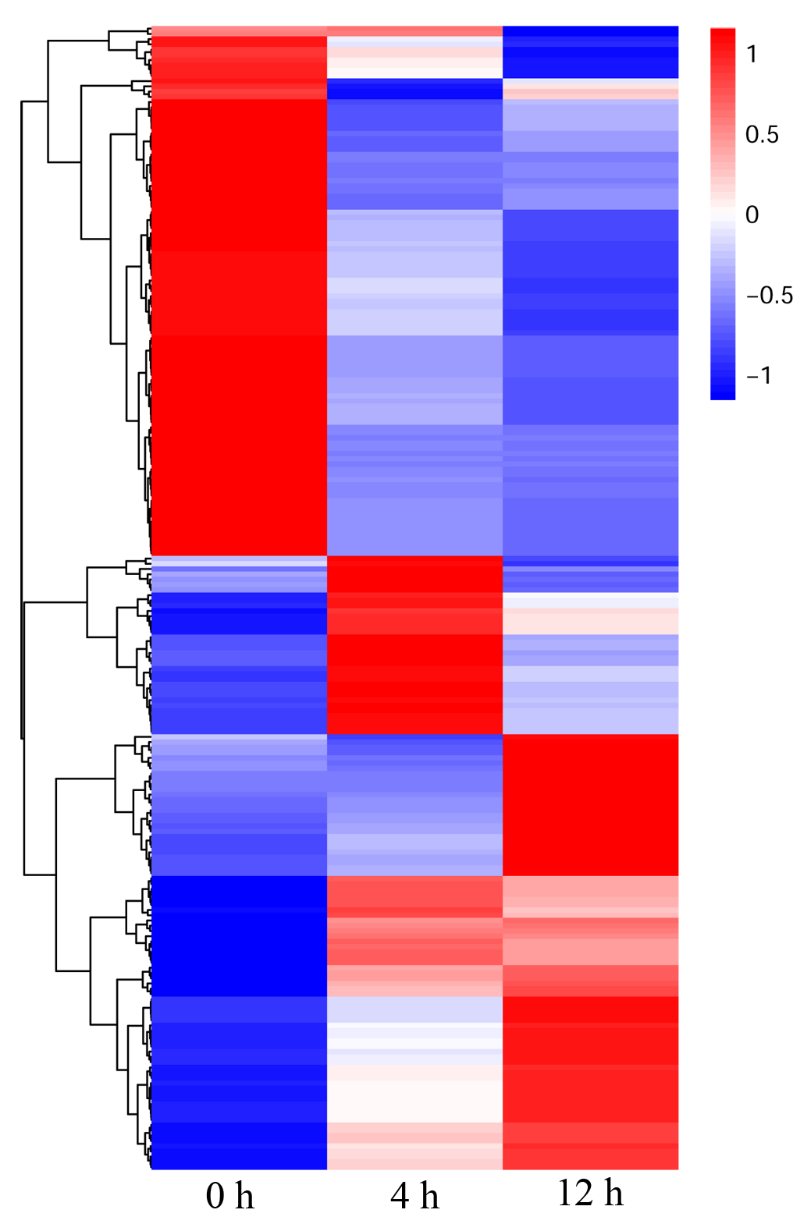


Supplementary Figure 3 Heatmap representing row normalization of FPKM values of DEGs involved in the amino acid metabolic pathway. The color scale represents the gene expression level: Red indicates high expression while blue indicates low expression. The heatmap was generated by R version 3.4.1 (https://www.r-project.org/) using the heatmap package.


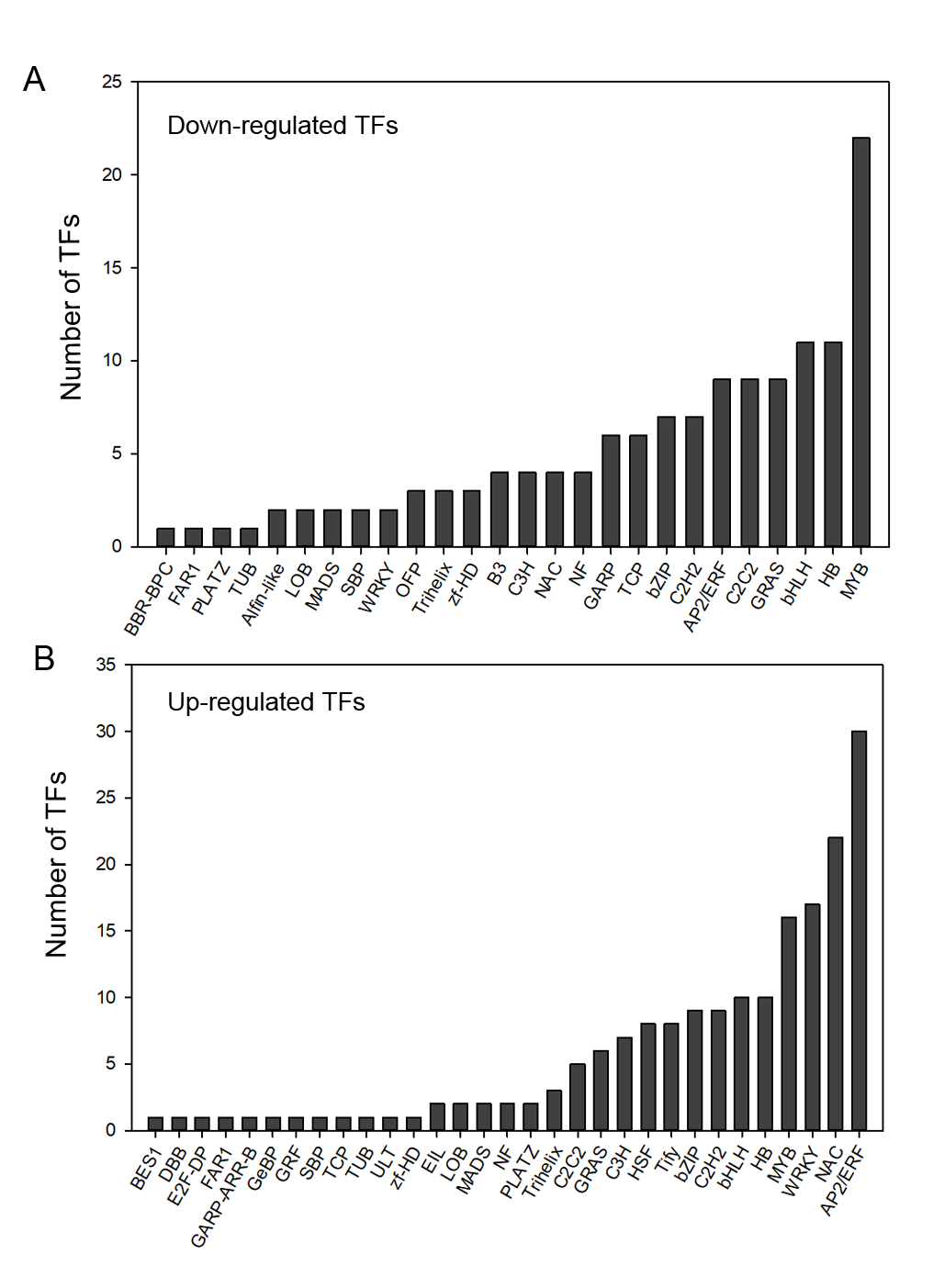


Supplementary Figure 4. Analysis of up-regulated transcription factors (TFs) and down-regulated TFs at both time points (4 h and 12 h after salinity stress) in *Dendrobium officinale* roots. (A) Down-regulated TFs at 4 h and 12 h. (B) Up-regulated TFs at 4 h and 12 h.


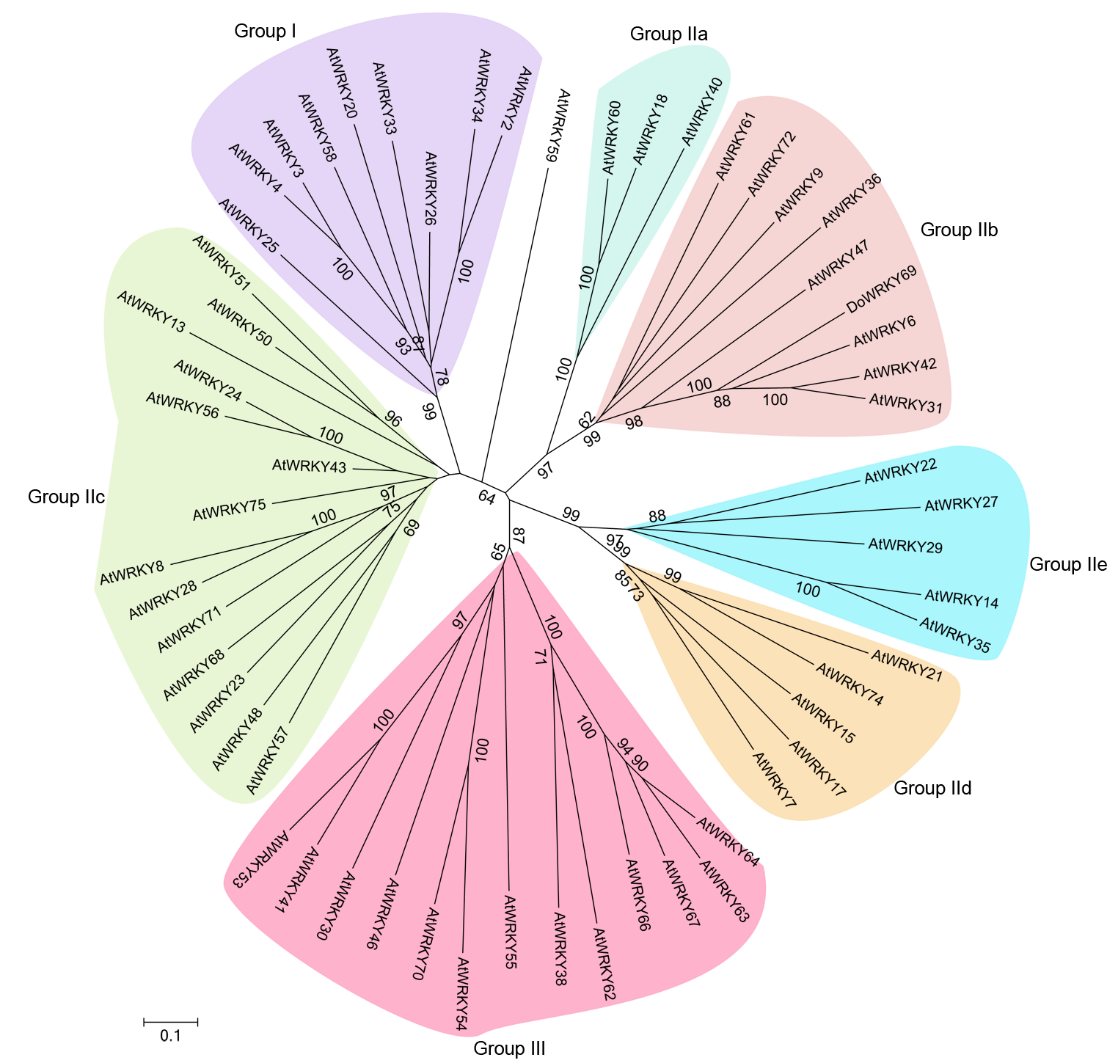


Supplementary Figure 5. Phylogenetic analysis of DoWRKY69 and *Arabidopsis thaliana* WRKY proteins. The DoWRKY69 protein and 58 AtWRKY proteins were aligned by MAFFT 7. The phylogenetic tree was generated by MEGA 7 using the Neighbor–Joining method with 1000 bootstrap replicates.





Supplementary Figure 6. Relative expression of *DoWRKY69* under salinity stress based on RNA-seq data.

Supplementary Table 1 Gene-specific primers for real time quantitative PCR.

| Primer name | Sequence |
| --- | --- |
| ActinF | 5'-TCCCAAGGCAAACAGAGAAA-3' |
| ActinR | 5'-GGCCACTAGCATATAGGGAAAG-3' |
| Do4CL-F | 5'-CCTCAAACTCCTCTCCATCAAC-3' |
| Do4CL-R | 5'-GTCAGCTGGTTCGGTAAGTAAG-3' |
| DoAOS-F | 5'-TGATGCGCCAGGAATCAT-3' |
| DoAOS-R | 5'-GAGAGTAGCGTCCTCGATAAAC-3' |
| DoCOMT-F | 5'-CATTCTGCCTGAAAGTCCAAAC-3' |
| DoCOMT-R | 5'-TCTGTCCTCTCTCTTCCACTT-3' |
| DoLOX2S-F | 5'-CACGCATAGAGAGCAGAATAGAG-3' |
| DoLOX2SI-R | 5'-CGGCATGAAATTGTGTAGCATAG-3' |
| DoOPR-F | 5'-GGGAGGATATGACAGAGAAGATG-3' |
| DoOPR-R | 5'-CGTCGAGGAAGATCAGGATTAG-3' |
| DoPsaDF | 5'-CGCCTTCGATCCAAATACAA-3' |
| DoPsaDR | 5'-TGACGGTAATCGGACTGACA-3' |
| DoPsaKF | 5'-CTCTGGCCTCAAAGCTCAAC-3' |
| DoPsaKR | 5'-ATGTGCCCAACTGTACCACA-3' |
| DoSAUR1F | 5'-TCAGATCTCGTTGACCACCA-3' |
| DoSAUR1R | 5'-AACCAGCTCATCAAGCGACT-3' |
| DoRBCS1F | 5'-CTCCGGCTCAGTCTACCTTG-3' |
| DoRBCS1R | 5'-TGCTTGAGCAAACGTTCATC-3' |
| DoSAUR2F | 5'-GGAGGAGGAGAGGAAGAGGA-3' |
| DoSAUR2R | 5'-GCATGGAAGTAAGGGAACGA-3' |
| DoGOT1F | 5'-TGGTTCACTTCGTCTTGCTG-3' |
| DoGOT1R | 5'-CGATCAGATCCGCAATTTTT-3' |
| DoWRKY69F | 5′-CCACATAACCTTGCCGATACA-3′ |
| DoWRKY69R | 5′-GATGCGTTCCGCCGATAATA-3′ |
| AtUBQ10F | 5′-GATCTTTGCCGGAAAACAATTGGAGGATGGT-3′ |
| AtUBQ10R | 5′-CGACTTGTCATTAGAAAGAAAGAGATAACAGG-3′ |

The primer pairs of DoWRKY69F/R and AtUBQ10F/R were used to detect the expression of *DoWRKY69* in transgenic lines and wild type plants. F, forward; R, reverse.

Supplementary Table 2 Statistics of paired-end sequence library for *D*. *officinale* under salt stress treatment.

| Samples | Clean bases | Clean reads | Mapped reads (%) | % ≥ Q30 |
| --- | --- | --- | --- | --- |
| Control-1 (0 h) | 6,375,341,448 | 42689286 | 0.8962 | 0.9365 |
| Control-2 (0 h) | 6,925,589,908 | 46365200 | 0.8968 | 0.9343 |
| Control-3 (0 h) | 7,539,143,846 | 50588120 | 0.9085 | 0.9355 |
| Salt treatment 1-1 (4 h) | 6,485,899,826 | 59782384 | 0.9074 | 0.9324 |
| Salt treatment 1-2 (4 h) | 7,297,589,620 | 48262238 | 0.9072 | 0.9333 |
| Salt treatment 1-3 (4 h) | 7,804,752,942 | 53156520 | 0.9092 | 0.9355 |
| Salt treatment 2-1 (12 h) | 8,899,359,736 | 43473460 | 0.9031 | 0.9329 |
| Salt treatment 2-2 (12 h) | 7,189,182,866 | 48920480 | 0.9043 | 0.9349 |
| Salt treatment 2-3 (12 h) | 7,944,454,968 | 52350578 | 0.9069 | 0.9358 |
